# Analysis of sea star larval regeneration reveals conserved processes of whole-body regeneration across the metazoa

**DOI:** 10.1186/s12915-019-0633-9

**Published:** 2019-02-22

**Authors:** Gregory A. Cary, Andrew Wolff, Olga Zueva, Joseph Pattinato, Veronica F. Hinman

**Affiliations:** Department of Biological Sciences, Carnegie Mellon University, Mellon Institute, 4400 Fifth Ave, Pittsburgh, PA 15213 USA

**Keywords:** Regeneration, Sea star, Echinoderm, Transcriptome

## Abstract

**Background:**

Metazoan lineages exhibit a wide range of regenerative capabilities that vary among developmental stage and tissue type. The most robust regenerative abilities are apparent in the phyla Cnidaria, Platyhelminthes, and Echinodermata, whose members are capable of whole-body regeneration (WBR). This phenomenon has been well characterized in planarian and hydra models, but the molecular mechanisms of WBR are less established within echinoderms, or any other deuterostome system. Thus, it is not clear to what degree aspects of this regenerative ability are shared among metazoa.

**Results:**

We characterize regeneration in the larval stage of the Bat Star (*Patiria miniata*). Following bisection along the anterior-posterior axis, larvae progress through phases of wound healing and re-proportioning of larval tissues. The overall number of proliferating cells is reduced following bisection, and we find evidence for a re-deployment of genes with known roles in embryonic axial patterning. Following axial respecification, we observe a significant localization of proliferating cells to the wound region. Analyses of transcriptome data highlight the molecular signatures of functions that are common to regeneration, including specific signaling pathways and cell cycle controls. Notably, we find evidence for temporal similarities among orthologous genes involved in regeneration from published Platyhelminth and Cnidarian regeneration datasets.

**Conclusions:**

These analyses show that sea star larval regeneration includes phases of wound response, axis respecification, and wound-proximal proliferation. Commonalities of the overall process of regeneration, as well as gene usage between this deuterostome and other species with divergent evolutionary origins reveal a deep similarity of whole-body regeneration among the metazoa.

**Electronic supplementary material:**

The online version of this article (10.1186/s12915-019-0633-9) contains supplementary material, which is available to authorized users.

## Background

**Fig. 1:**
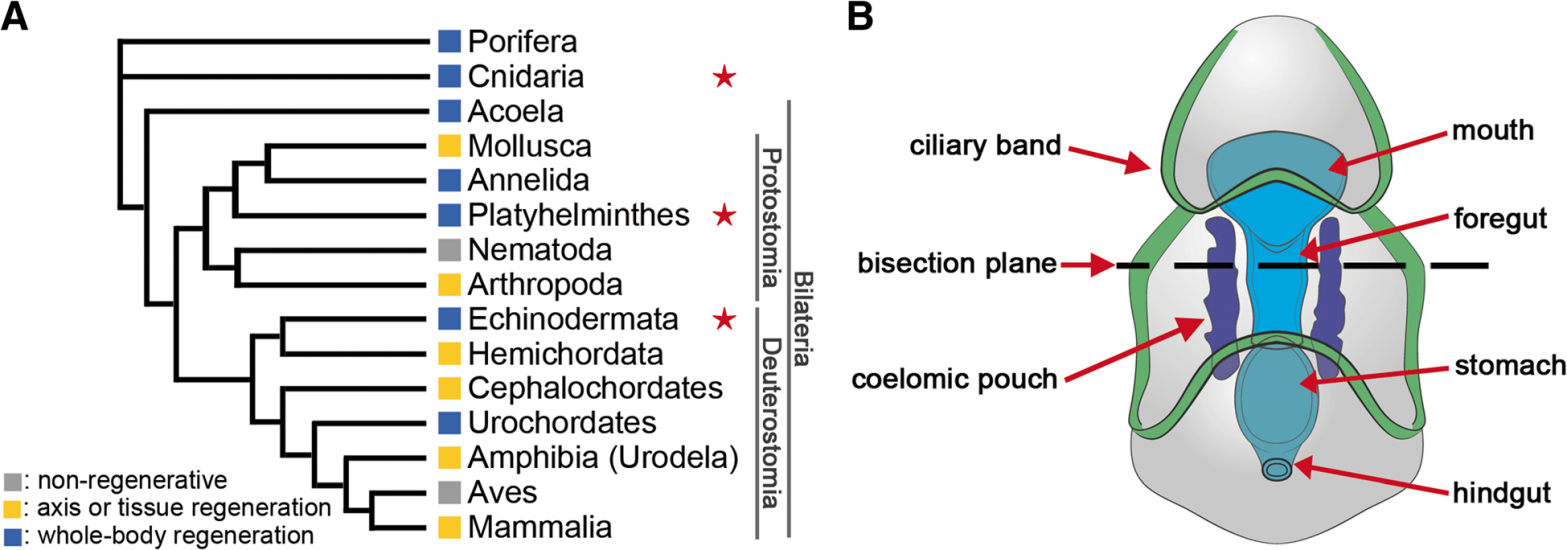
Models of whole-body regeneration**. a** Phylogeny depicting regeneration capacity of various taxa, after [2, 89]. Species from the three taxa marked with a star were considered in this study. **b** Schematic of a sea star bipinnaria larva indicating the bisection plane (dashed line) and relevant anatomical features including the ciliary band epithelium (green), coelomic pouch epithelium (purple), and enteric organs (blue)

The evolution of regenerative abilities has fascinated researchers for centuries. Species with a capacity for restorative regeneration are distributed throughout the metazoan tree of life (Fig. 1a); however, the extent to which any animal is capable of regenerating varies considerably. Whereas some taxa are able to undergo whole-body regeneration (WBR), other lineages exhibit much more restricted regenerative capabilities (e.g., the ability to re-grow only specific organs or tissues) [1–3]. Given the broad phylogenetic distribution of robust regenerative abilities, it remains unclear if elements of this phenomenon are directed by deeply conserved molecular mechanisms that have been lost in species with more restricted regenerative capacities or have evolved multiple times independently. While many attempts have been made to synthesize regenerative phenomena in disparate taxa [1–3], or to provide evolutionary context to genes utilized during regeneration within a particular model [4, 5], few studies have directly compared the transcriptional control of regeneration among highly regenerative, distantly related metazoan lineages. This is, in part, because we are as yet missing detailed descriptions of regeneration from key taxa. By approaching regeneration from an evolutionary perspective, it is possible to identify shared mechanisms that underlie regenerative abilities. This has significant implications for if and how regeneration can be induced in organisms with more limited potential.

The best characterized models for understanding regeneration are species of Cnidaria (e.g., *Hydra vulgaris* [6, 7]) and planaria (e.g., *Schmidtea mediterranea* [8, 9]). These organisms are capable of WBR, meaning that they can re-grow all body parts following amputation [2]. In these contexts, WBR involves transitions through wound healing, immune signaling, axis/organizer specification (especially via WNT signaling), cell proliferation, and differentiation of new cells to replace missing cells and tissues [7–11]. A key distinction between these models lies in the source of the newly differentiated cells. In planarians (bilaterian protostomes within the phylum Platyhelminthes), a pool of somatic stem cells (neoblasts) generates a proliferative blastema that is essential for regeneration [12–14]. In contrast, regeneration in *Hydra* species is mediated through de-differentiation and transdifferentiation of existing cells to replace those lost by injury [15, 16], in addition to somatic stem cells (interstitial cells or I-cells), which serve as both undifferentiated precursors of several cell types [17] and also proliferate following injury [18].

Regenerative ability is generally more limited in deuterostomes. Within vertebrates, regeneration is frequently restricted to specific developmental stages, tissues, or organs [2]. By contrast, many invertebrate deuterostomes are capable of extensive regeneration of all tissues at multiple developmental stages. Colonial ascidians (e.g., *Botryllus schlosseri*) are capable of WBR [19, 20], whereas solitary species are capable of partial regeneration (e.g., adult siphons in *Ciona intestinalis*) [21, 22]. Hemichordate species (e.g., *Ptychodera flava*) can regenerate the adult head when bisected from the body [23, 24]. However, the best known and most regenerative species of deuterostomes belong to the Echinodermata.

Echinoderms (e.g., sea stars, brittle stars, and sea cucumbers) exhibit remarkably robust regenerative capabilities throughout all life stages. Adult echinoderms have been a focus of regeneration studies that examined re-growth of specific structures (e.g., spines, tube feet, nerve cord, gut, and arms) [25–39]. Regeneration has also been observed in larvae from all echinoderm classes examined [40]. These planktonic stage echinoderms can swim and feed in the water column for weeks or months. Larval regeneration is more similar to the WBR observed in planaria and hydra, as it requires the complete re-growth of all tissues and organ systems. Molecular studies of regenerating sea star larvae have identified several regeneration-specific changes in gene expression, including the *sea star regeneration-associated protease* (SRAP; [41]), *vasa*, *nodal*, *dysferlin*, and *vitellogenins* (*vtg1* and *vtg2*) [42]. However, to date, a comprehensive survey of gene expression changes during larval echinoderm regeneration has not been reported. As one of the few deuterostome taxa capable of undergoing WBR, sea star larvae can provide unique insight into the evolution of regenerative processes.

Here, we characterize the molecular and cellular events that occur during regeneration in the larval sea star *Patiria miniata* and assess the expression patterns of orthologous genes in other distantly related species that undergo WBR. We first characterize the landmark regeneration events: wound healing, tissue re-proportioning, cellular proliferation, and cell death. To characterize the transcriptional changes that underpin these events, bisected larval fragments were evaluated using RNA-Seq. Through analysis of these data, we define broad gene classes that are expressed similarly in both anterior and posterior regenerating fragments. Finally, through identification of orthologous genes between *P. miniata* and published datasets of regenerating hydra and planarian models (Fig. 1a), we find sets of genes that have similar temporal expression profiles in these distantly related regenerating organisms. These results highlight similarities in the regeneration programs of a bilaterian deuterostome, a lophotrochozoan, and a basally branching eumetazoan. This suggests that WBR may be common to the base of all animals.

## Results and discussion

### Bipinnaria regeneration involves wound healing, body re-proportioning, cell proliferation and cell death

To make an informed comparison to other regenerative models, we first characterized the stages of larval regeneration in *P. miniata*. Bipinnaria larvae (7 days post-fertilization [dpf]) were bisected midway along the transverse anterior-posterior (AP) axis (Fig. 1b). Both resulting larval fragments were completely regenerative, restoring all lost tissues and organs over the course of 2 weeks. These findings are consistent with previous reports of larval sea star regeneration [42, 43]. Although we focus on the regeneration of the posterior fragments, a similar regenerative response is apparent within the anterior fragment (Additional file 1: Figure S1).

**Fig. 2:**
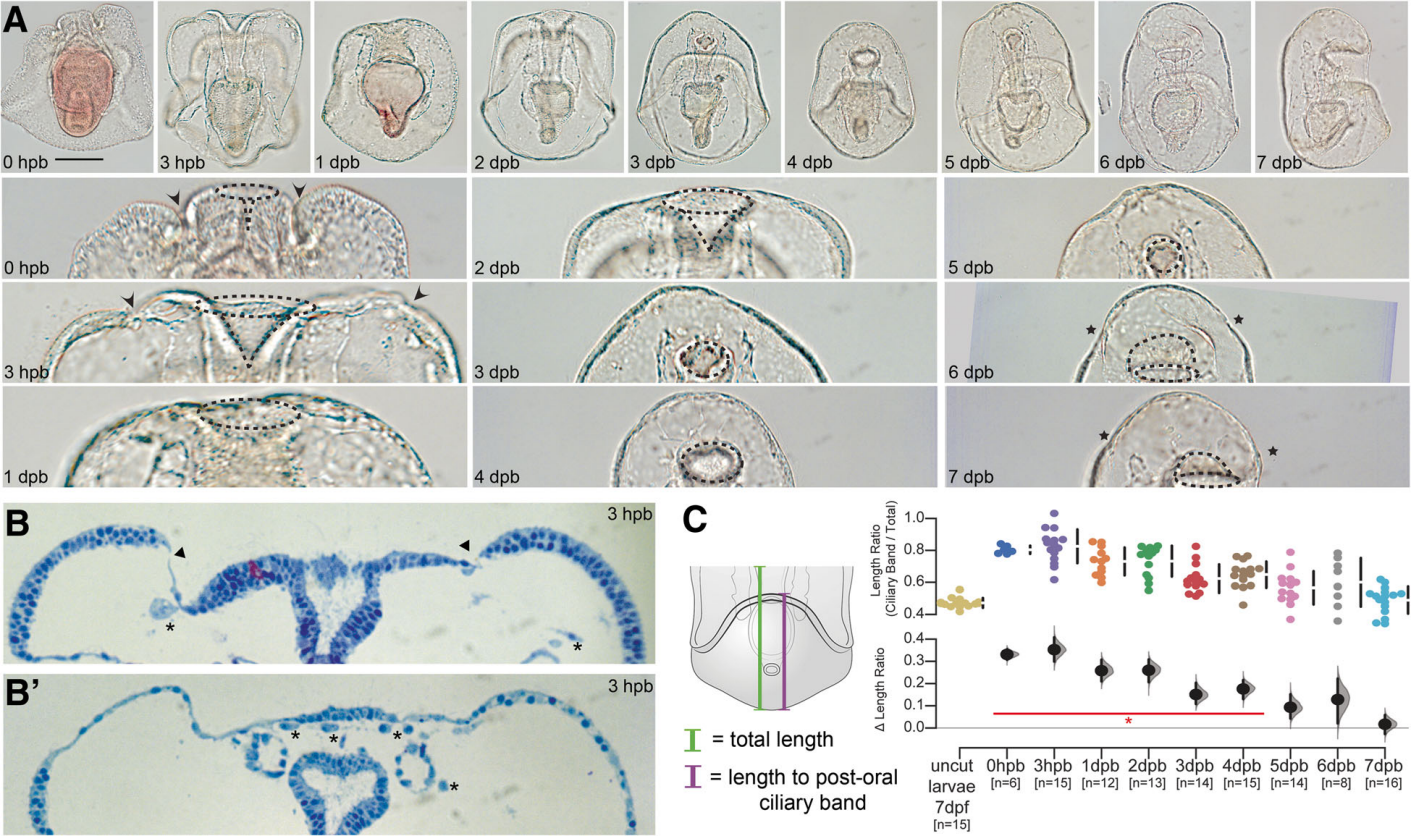
Sea star bipinnaria regeneration involves wound healing, re-proportioning, and respecification.(**a**) DIC images showing larval recovery following bisection (top row) and magnifications of the wound site at each stage (bottom row). Important anatomical features are highlighted in the magnified images including the wound site (arrowheads), opening to the gut lumen (dotted lines), and new ciliary bands (asterisks). Scale bar = 100 μm; applicable to all images in panel. (**b**) Two serial sections from the same individual showing wound closure (arrowheads) and many free cells within the blastocoelar space (asterisks). (**c**) Ratios of length from the posterior pole to the top of the post-oral ciliary band to length from the posterior pole to the anterior pole (i.e., total length of the specimen) are plotted along with the difference of the means (i.e., Δ length ratio) and 95% confidence interval. Those timepoints with a ratio found to be significantly different than uncut larvae are indicated by the red line and asterisk (Mann-Whitney *U* test, *p* value < 0.001). *n* = number of individuals measured at each timepoint

We observe that the initial wound is mostly closed by 3 h post-bisection (hpb; Fig. 2a, b, arrowheads). This also coincides with the appearance of several types of mesenchymal blastocoelar cells proximal to the wound epithelium. After this rapid wound healing response, larvae re-proportion their remaining tissues over the first several days post-bisection (dpb). This is evident when analyzing the position of the post-oral (lower) ciliary band (Fig. 2c). Prior to bisection, these ciliary bands are located in the middle of the larva; on average, the distance from the posterior end of the larva to the ciliary band is 47% of the total length of the larva (Fig. 2c). Immediately after bisection, this ratio increases to 80% as the anterior region has been removed (Additional file 1: Figure S2). However, over the subsequent 5 days, the larval proportions return to pre-bisection ratios (at 5 dpb, the ciliary band to larval length ratio is 57%). Importantly, this reallocation of tissues is not due to an increase in the total length of the larval fragments, as we show that the overall length of the bisected larva does not change during this time (Additional file 1: Figure S2). Although we did not quantify the change, we note a similar re-proportioning of the larval midgut between 1 and 5 dpb and also observed that the shape and position of the larval mouth changes. During bisection, the foregut is cut in half such that the anterior portion forms a new oral opening oriented along the anterior-posterior axis. However, by 3 dpb, the oral opening is reoriented ventrally and tissues are apparently anterior to this opening. Finally, by 6 dpb, we observe the return of most morphological features, including the anterior ciliary band, the oral field, and oral lobe. Together, these findings indicate that regeneration in larval sea stars occurs in at least three stages: healing at the wound site, re-proportioning the remaining tissues, and restoration of lost tissue. Similar patterns are evident in regenerating anterior fragments (Additional file 1: Figure S2).

**Fig. 3:**
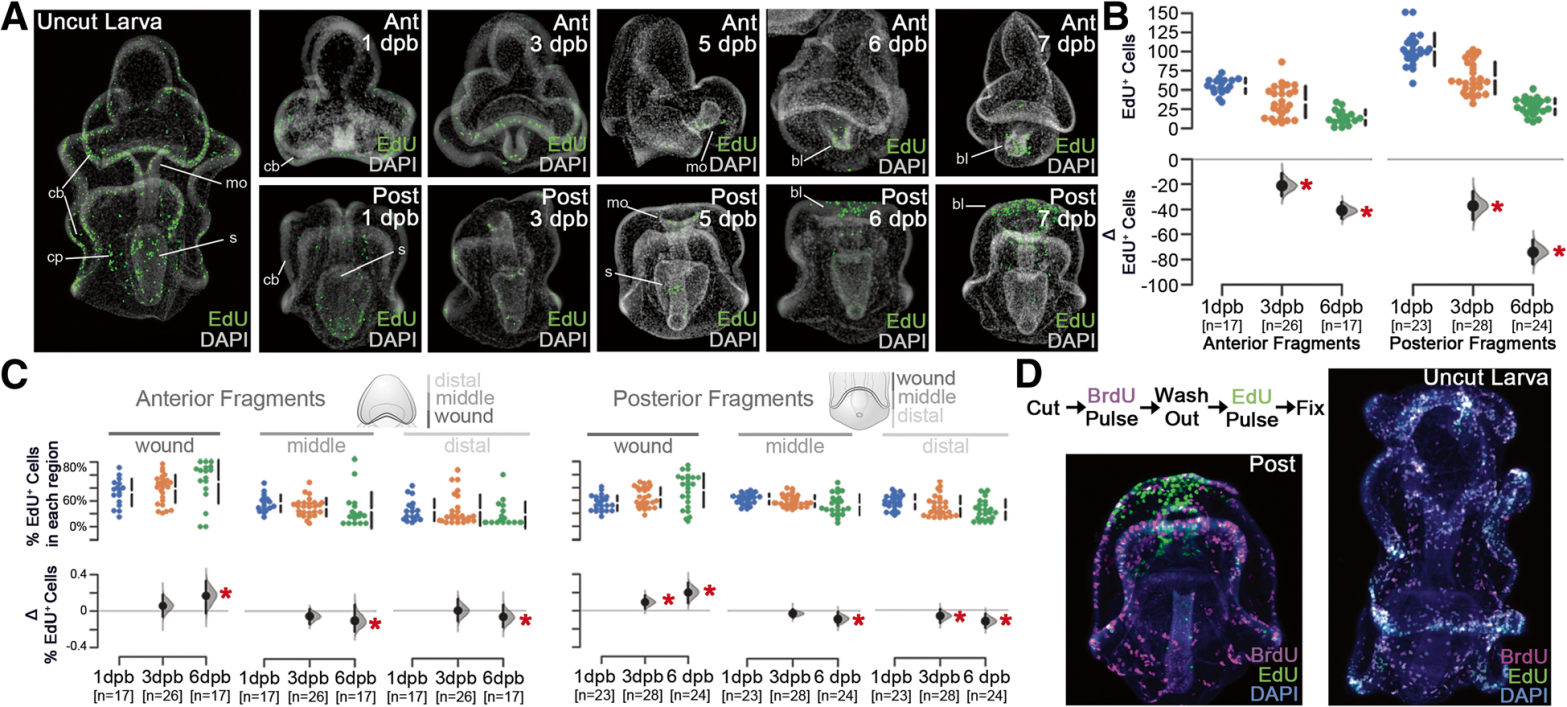
Cell proliferation decreases and localizes to wound-proximal cells. **a** EdU staining of S-phase cells in intact and regenerating sea star larvae (1–7 days post-bisection [dpb]). EdU-positive cells are shown in green. Nuclei were stained with DAPI and shown in gray. Cell proliferation in uncut larvae is throughout the ciliary band epithelium (cb), mouth (mo), stomach (s), and coelomic pouches (cp). Regenerating anterior fragments (top row) and posterior fragments (bottom row) demonstrate similar initial distributions of proliferation, although the number of EdU^+^ cells decreased by 3 dpb. Beginning at 6 dpb, EdU^+^ cells are concentrated near the wound site in both anterior and posterior regenerating fragments in a putative regeneration blastema (bl). **b** Quantitation of the EdU^+^ cells shows a steady decline in the number of proliferating cells in both anterior and posterior regenerating fragments. The difference of the means (i.e., Δ EdU^+^ Cells) is plotted and significance differences are indicated (Mann-Whitney, *p* < 0.05, red asterisk). *n* = total number of bisected animals counted. **c** The fraction of EdU^+^ cells in each of the wound-proximal, middle, and wound-distal thirds of each regenerating larval fragment from panel B is shown. The number of individuals counted is the same as in (**b**). The difference of the means (i.e., Δ % EdU^+^ cells) is plotted and significance differences are indicated (Mann-Whitney, *p* < 0.05, red asterisk). **d** The experimental regimen of the BrdU/EdU pulse-chase experiments is shown. Regenerating larvae (left) or uncut larvae (right) were labeled with BrdU (magenta) for 6 h after which the BrdU was washed out. Larvae are subsequently labeled with a 6 h EdU pulse (green) at the onset of wound-proximal proliferation or after a similar duration for uncut larvae

We next analyzed the pattern of cellular proliferation during regeneration. Larvae were exposed to EdU (6 h pulses) to mark proliferating cells in normal (uncut) and over the course of larval regeneration (Fig. 3). In uncut larvae, EdU^+^ cells are widely distributed (Fig. 3a). We infer from this result that larvae are actively growing. However, upon bisection, the numbers of EdU^+^ cells steadily decrease (Fig. 3b; Mann-Whitney *P* < 2 × 10^−4^). This decrease in EdU^+^ cell number is accompanied by a change in the localization of proliferating cells. EdU^+^ cells localize proximally to the wound sites (3 dpb in posterior fragments and 6 dpb in anterior fragments), and fewer EdU^+^ cells are located in more distal tissues distal (Fig. 3c; Mann-Whitney *P* < 0.05). Moreover, the proliferating cells that localize to the wound site are distinct from cells that proliferate early. Cells proliferating at 1 dpb were labeled with pulse of BrdU followed by a wash-out. Cells proliferating during the later phases were then labeled with a pulse of EdU and processed for imaging. We find very little overlap of BrdU^+^ cells that are also EdU^+^ (Fig. 3d). This indicates that cells proliferating during early regeneration do not to continue to divide during the later, wound-proximal proliferation phase of regeneration. In non-bisected, stage equivalent control larvae, by contrast, there is extensive overlap between BrdU^+^ and EdU^+^ cells (Fig. 3d). This suggest that under normal conditions, cells that are proliferating normally continue to divide, but following bisection, different populations of cells now enter proliferation. Thus, during the regenerative response, typical, system-wide larval growth is inhibited, and regeneration-specific cell proliferation is concentrated at the regenerating edge where tissues later form.

**Fig. 4:**
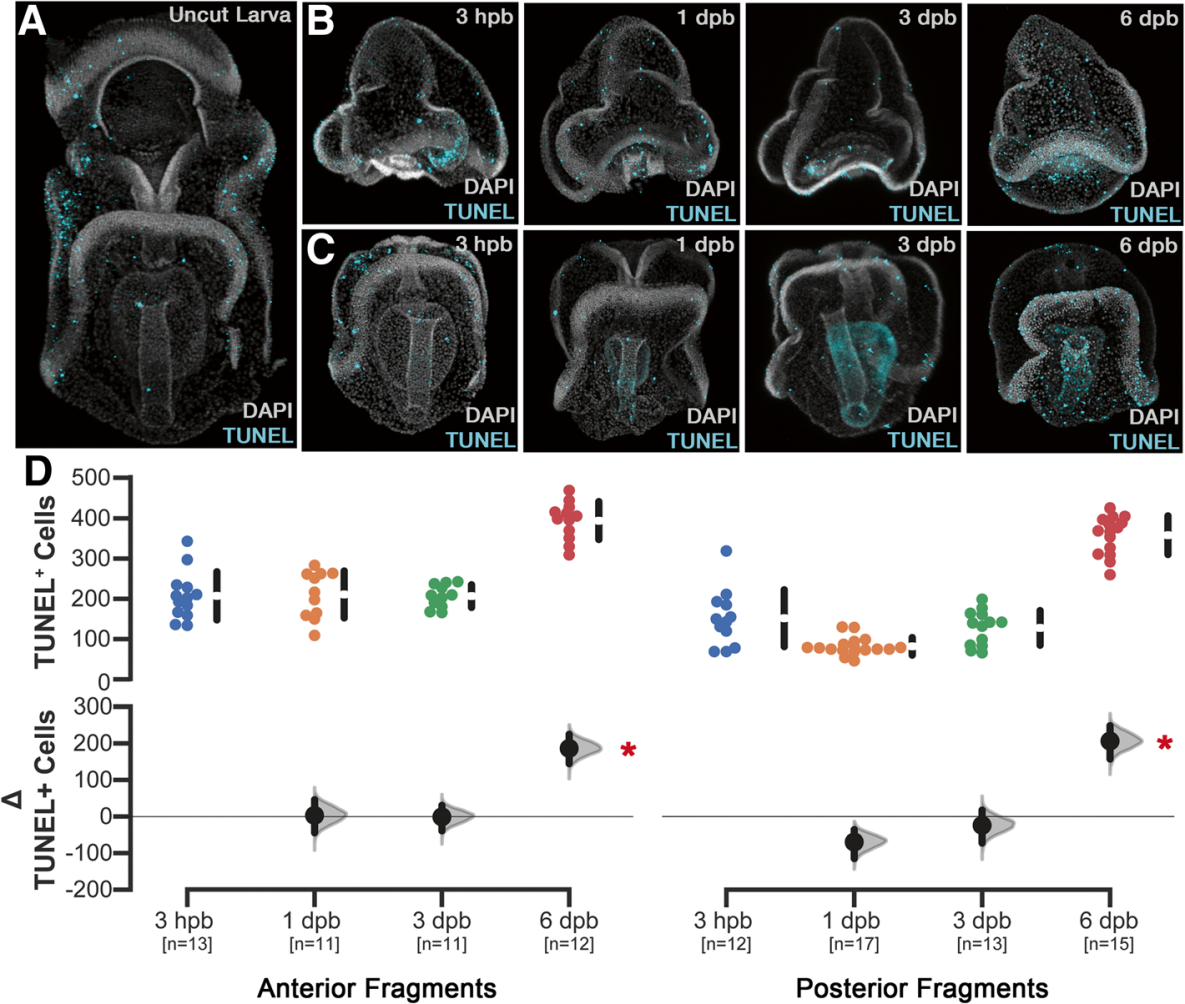
Apoptotic cell death persists and increases in later phases. **a** TUNEL^+^ cells (green) in control animals are normally distributed throughout larval tissues and is concentrated within the ciliary band epithelium. Nuclei (gray) stained with DAPI. Regenerating anterior (**b**) and posterior (**c**) fragments display similar patterns and numbers of TUNEL^+^ cells from 3 h post-bisection (hpb) until 6 days post-bisection (dpb) when there is an increase. **d** Quantitation of TUNEL^+^ cells in regenerating anterior and posterior fragments shows that there is no significant difference in the number of TUNEL^+^ cells until 6 dpb when a significant increase in apoptotic cells are detected. The difference of the means (i.e., Δ TUNEL^+^ cells) is plotted and significance differences are indicated (Mann-Whitney, *p* < 3 × 10^−4^, red asterisk). *n* = the number of individuals sampled

As a corollary to understanding cell division during larval regeneration, we examined the patterns of cell death using TUNEL assays. In normal larvae, TUNEL^+^ cells are distributed organism wide (Fig. 4a). Following bisection, the number and distribution of apoptotic cells remains largely unchanged for several days (Fig. 4b–d and Additional file 1: Figure S3). However, at 6 dpb, there is a significant increase in the total number of TUNEL^+^ cells in both anterior and posterior regenerating fragments (Mann-Whitney *P* < 4 × 10^−5^). Unlike cell proliferation, these cells are not preferentially located with respect to the wound epithelium (Additional file 1: Figure S3B). Together, these results indicate that regeneration induces a global decrease in cell proliferation, followed by a rapid increase in cells cycling near the wound site. In contrast, the rate of cell death is consistent and increases across the larva coincident with the onset of wound-localized cellular proliferation.

These cellular and tissue changes during larval sea star regeneration define landmark features of the regenerative process including wound healing, re-proportioning of larval tissues, and onset of wound-proximal proliferation along with a coincident increase in apoptotic cell death. These broad characterizations mirror regenerative processes described in other organisms and suggest a shared toolkit of regenerative responses.

### Transcriptome analyses of larval regeneration explain the genetic basis underlying observed cellular and morphological phenomena

To characterize the molecular events that operate during larval sea star regeneration and to establish a dataset amenable to inter-species comparison, we surveyed gene expression changes across a time course of larval regeneration. Pools of regenerating posterior fragments, anterior fragments, and non-bisected sibling control larvae were collected at three points following bisection: one early time point (approximately 3 hpb), one intermediate time point (3 days post-bisection, dpb), and one time point at the initiation of wound-localized cell proliferation (6 dpb). By separately sampling RNA from each pool of regenerating fragments, we were able to identify changes in gene expression changes that occur in both the anterior and posterior fragments as well as those that are specific to regeneration in each context. The inclusion of non-bisected, age-matched, sibling larvae control for transcriptional changes due to continuing larval development as well as genetic differences among cultures. For each time point, transcript levels were compared between each pool of regenerating fragments and the control larvae (i.e., anterior vs. uncut and posterior vs. uncut). In total, 9211 differentially expressed genes (DEG) were identified from these comparisons (Additional file 2: Table S1).

**Fig. 5:**
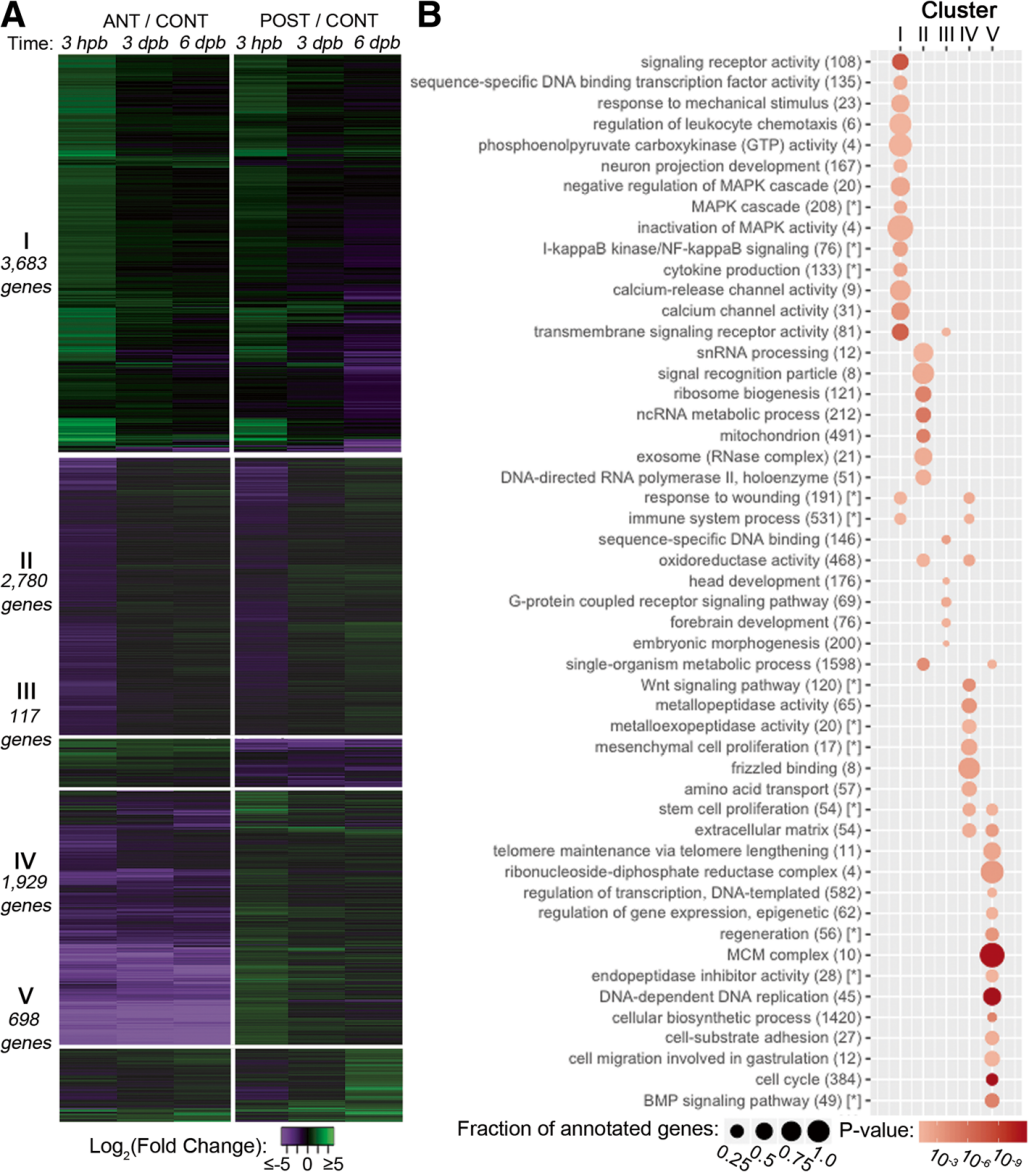
Cluster analysis indicates genes involved in regenerative functions. **a** The heatmap depicts log fold change values for genes (rows) in anterior (ANT) and posterior (POST) regenerating fragments compared with sibling uncut control (CONT) larvae over the sampled regeneration time points (columns; 3 h post-bisection [hpb], 3 days post-bisection [dpb], and 6 dpb). Green indicates a positive fold change (upregulated with respect to uncut controls), whereas purple indicates a negative fold change (downregulated with respect to control). **b** Gene ontology (GO) term enrichments for each of the five clusters. The enrichment of each GO term is indicated by a circle where the area corresponds to the fraction of genes annotated with that term are present in the cluster, and the color of the circle corresponds to the corrected hypergeometric *p* value of term enrichment. Terms marked with an asterisk [*] are from the annotation set generated by mouse gene ortholog prediction (Fig. 5, Additional file 1: Figure S3)

We implemented a hierarchical clustering approach to distinguish fragment-specific expression patterns from expression changes that are shared in both regenerating fragments (Fig. 5a and Additional file 1: Figure S4). In total, five expression clusters were identified: (I) genes upregulated early in both anterior and posterior fragments, (II) genes downregulated early in both fragments, (III) genes up in the anterior and down in the posterior, (IV) genes up in the posterior and down in the anterior, and (V) genes upregulated later (i.e., by 6 dpb) in both fragments (Fig. 5a). Thus, we have identified three subsets of DEGs that exhibit similar expression profiles during regeneration in both fragments (i.e., clusters I, II, and V) and two subsets that are strongly fragment-specific (i.e., clusters III and IV). To validate the RNA-Seq measurements, we analyzed the same samples using a custom Nanostring nCounter codeset. In total, 69 of the 74 genes (92.3%) tested by our Nanostring experiments exhibited either a similar trend and significance status or just a similar trend to the measurements made by RNA-Seq (Additional file 1: Figure S5).

To provide further insight into the functions of genes that were assigned to each cluster, we identified enriched Gene Ontology (GO) terms (Fig. 5b and Additional file 1: Figure S6). Genes in clusters I and II (i.e., genes that are up- or downregulated early in both regenerating fragments) are enriched for GO terms associated with a robust wound response. Upregulated genes (cluster I) are enriched for terms that include cell signaling pathways (e.g., “MAPK cascade” and “calcium channel activity”), “response to wounding,” and “immune system process” (Fig. 5b and Additional file 1: Figure S6). This cluster is also enriched for terms that indicate an early involvement of innervation and ciliogenesis (e.g., “neuron projection development” and “motile cilium”) which are common in other regeneration models [44–47]. The downregulated genes (cluster II) are enriched for terms that point to a shut-down of anabolic processes (“ribosome biogenesis” and “gene expression”) as well as primary metabolism (e.g., “mitochondrion” and “metabolic process”). Together, these clusters of early-regulated genes are consistent with a rapid response to the bisection insult that involves downregulation of highly energetic cellular processes and upregulation of functions that are specific to the injury response.

Clusters III and IV are composed of genes whose profiles are highly fragment-specific; these genes are differentially regulated in each fragment relative to control larvae. Many of these genes are expressed asymmetrically along the AP axis. Thus, bisection results in the loss of posterior-specific gene expression from anterior fragments and vice versa. For example, cluster III is enriched for genes annotated with functions specific to anterior larval fragments, such as “head development” [48], whereas cluster IV is enriched for genes associated with posterior fates in embryonic sea stars, such as “Wnt signalling pathway” [49].

Finally, although cluster V is comprised of relatively few genes, it is the most functionally coherent cluster. That is, the GO term enrichment analyses are the most statistically significant and reproducible across the three sources of functional annotations tested, i.e., de novo annotations and annotations based on orthology to *Strongylocentrotus purpuratus* and *Mus musculus* (Fig. 5b and Additional file 1: Figure S6). Genes assigned to cluster V are enriched for terms related to the cell cycle, DNA replication, and extracellular matrix (ECM) remodeling. The cluster V genes, which are upregulated late (by 6 dpb) in both fragments, likely reflect the onset of localized cellular proliferation that occurs at this time (Fig. 3a). Importantly, these genes are upregulated in regenerating fragments although the total number of proliferating cells has decreased compared to controls (Fig. 3a). This suggests that the cluster V genes represent a regeneration-specific increase in expression of proliferation-associated genes that is distinct from the normal, growth-associated proliferation.

### Comparative transcriptome analyses reveal homologous genes with shared expression profiles among distantly related animals

Having identified the overall morphological progression of larval sea star regeneration (i.e., wound response, axis re-proportioning, and cell proliferation), we sought to determine if orthologous genes with similar temporal expression exist in other models of WBR. Such homology could indicate not only a shared overall progression, but that the genes involved are also in common. To address this question, we used published transcriptome data from regenerating planaria (*S. mediterranea*) [4] and hydra (*H. magnipapillata*) [5] for comparison. The Kao et al. dataset [4] was selected because it consolidated several planarian transcriptome assemblies, resulting in a more complete gene set, and also independently sampled both regenerating anterior and posterior worms, which is analogous to our own study design. Furthermore, the time points sampled range from 0 h post-amputation (hpa) to 72 hpa, at which point planarian blastemal proliferation reaches its peak [9]. This time frame roughly corresponds to the phases of regeneration considered in our study of larval sea stars. Regeneration has been less well characterized from a molecular standpoint in hydroids; the Petersen et al. dataset [5] is the only available transcriptome study from regenerating hydra. Here, RNA was sampled only from the distal tip of regenerating aboral tissues during the 48 h it takes to achieve complete head regeneration. As blastemal proliferation is not a feature of hydra regeneration, this characteristic cannot be used to synchronize the regenerative phases in this study to the other datasets. Nonetheless, these published datasets provide the best available basis for comparison to our sea star dataset.

To identify orthologs that share similar temporal dynamics during regeneration, the reported expression values from each dataset were clustered. For each comparative dataset, we assigned genes to three coarse clusters: those that were upregulated early in regeneration and downregulated later, those that were downregulated early in regeneration and upregulated in later regeneration, and those that exhibited some other temporal dynamic (Additional file 1: Figure S7 and S8). Finally, we identified genes in each of the five sea star expression clusters with orthologs in each of the planaria and hydra clusters. Using this approach, we find statistically significant overlaps between genes differentially expressed early in all three datasets as well as genes in the posterior-specific sea star cluster with clusters indicating fragment specificity in each of the other organisms. In the following sections, we describe how this allowed us to identify not only broad groupings of shared expression patterns but also specific orthologs similarly expressed across regeneration in these metazoans.

### Early features of the regenerative response are highly similar

By analyzing the kinetics of orthologous gene activity in WBR, we find the strongest correlation among genes that are differentially expressed early in each dataset. That is, a significant number of orthologs are upregulated at early regenerative stages in both the sea star and planaria, as well as the sea star and hydra datasets (hypergeometric *p* = 4.5 × 10^−3^ and *p* = 8.8 × 10^−9^, respectively; Additional file 1: Figure S7 and S8). This set of genes is enriched for GO terms that include “cilium,” “calcium transport,” and “signaling.” Similarly, we also found a significant number of orthologs are downregulated in response to bisection in both sea star and planaria (hypergeometric *p* = 3.3 × 10^−4^). These orthologs are enriched for GO terms such as “ncRNA processing” and “ribosome,” suggesting that early repression of the energetically expensive process of ribosome biogenesis is a fundamental element of WBR.

Two intracellular signaling pathways, Ca^2+^ mobilization and MAPK signaling, have been broadly implicated in wound response [50–54] and are found to be upregulated early in bipinnaria regeneration. Recent proteomic data indicate that calcium signaling is involved in the anterior regeneration in planaria [55]. MAPK signaling, through both ERK and JNK pathways, is important in neoblast control and blastema differentiation in planaria [56, 57], and JNK signaling has been specifically linked with restoration of proper axial patterning in planaria by re-activation of appropriate WNT signaling [58]. Studies in hydra have similarly demonstrated that wound-responsive MAPK signaling is necessary for early specification of the head organizer, and thus functional regeneration. Early MAPK signaling may thus be shared feature of highly regenerative organisms [59].

**Fig. 6:**
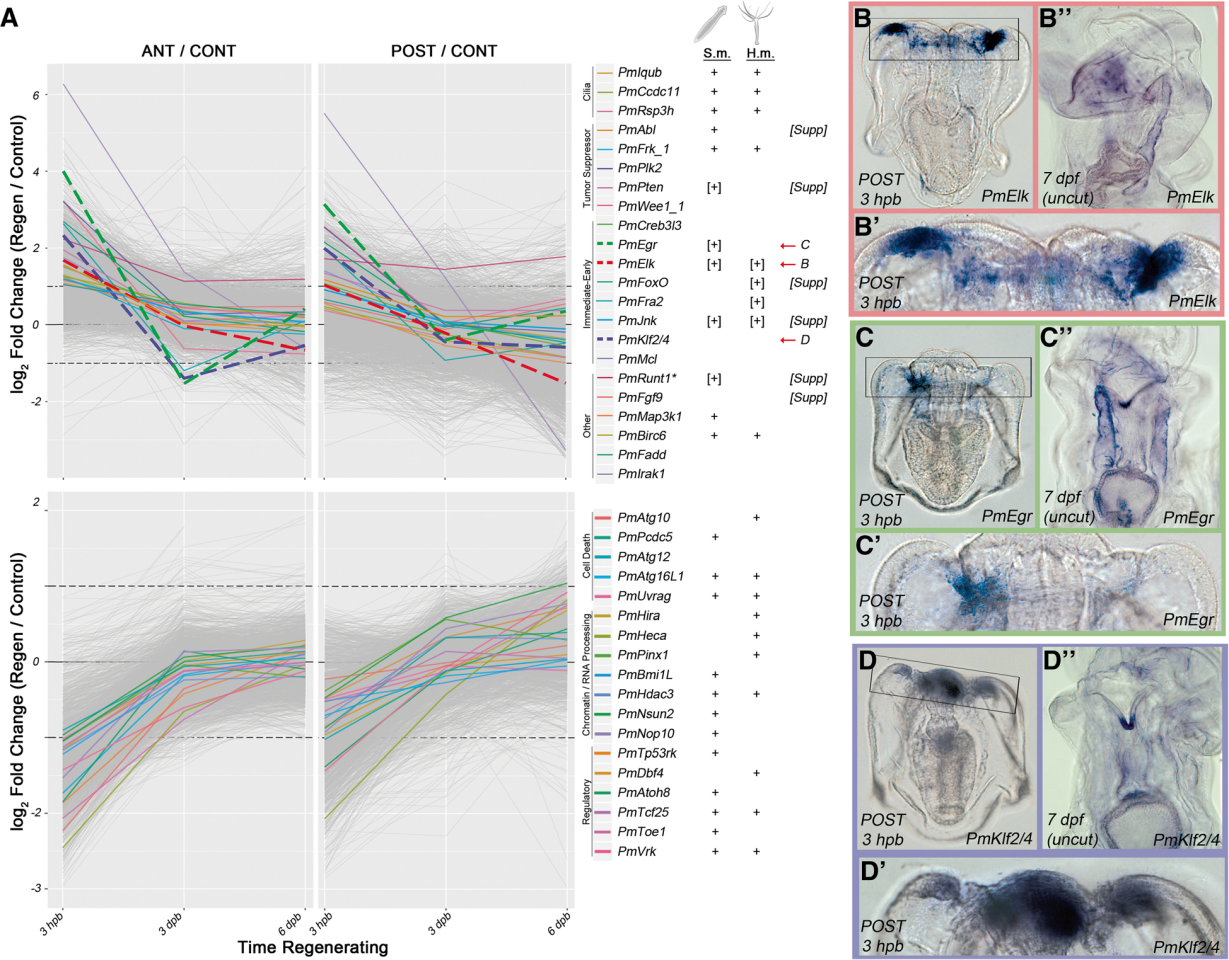
Evolutionarily similar early regeneration response. (**a**) These plots show sea star gene log fold change values for genes differentially expressed early in both anterior and posterior regenerating fragments compared with non-bisected sibling control larvae. Genes upregulated in both fragments (top row) correspond to cluster I, and genes downregulated in both fragments (bottom row) correspond to cluster II. All genes assigned to each cluster are plotted in gray. Several genes, either referenced in the text or representative of functions considered, are indicated with colored lines. Next to the key for each gene is an indication (+) of whether an ortholog for that gene was found in an analogous cluster in either the planaria (*S.m.*) or hydra (*H.m.*) datasets. Indicators in brackets (e.g., “[+]”) are those was no overlapping ortholog identified by our analyses, but genes with the same name were implicated by published datasets. Genes plotted with dashed lines are shown by in situ (right). Several additional genes are shown in a supplemental figure (Additional file 1: Figure S9). The expression patterns of *Elk* (**b**), *Egr* (**c**), and *Klf2/4* (**d**) are shown. (**b′**–**d′**) are magnifications of the wound site shown in the boxed regions in panels (**b**–**d**). Expression patterns in uncut larva are also shown (**b″**–**d″**)

The genes upregulated early in regeneration are also enriched for cilium-associated functions. The activation of these genes (e.g., *Ccdc11*, *Rsph3*, *Iqcd*, and *Iqub*; Fig. 6a) indicates that, in all three models, cilia play a central role in early regeneration. While this feature has not been reported in either planaria or hydra, a role for cilia in wound response and regeneration has been observed in mammals [45], zebrafish [47], and a related cnidarian (*Nematostella vectensis*) [46].

The set of similarly early activated genes also includes several key regulatory genes including orthologs of several tumor suppressor genes (i.e., *Abl*, *Menin*, *Frk*, *Pten*, *Rbbp6L*, *Plk2*, and *Wee1*; Fig. 6a). Several of these are also upregulated early in other regeneration models [60, 61]; these findings present an additional context in which the tumor suppressor genes show activity during regeneration. In regenerating sea star larvae, normal cell proliferation ceases prior to the emergence of the distinct wound-proximal proliferation (Fig. 3). The coincident activation of tumor suppressor genes and downregulation of ribosome biogenesis genes may be associated with this response. There is also an early signature of general cell cycle arrest in the hydra transcriptome [5]. While planarian neoblasts continue to proliferate at sites distal to the injury even during blastemal proliferation, inactivation of planarian *PTEN* gene homologs resulted in defective regeneration due to neoblast hyperproliferation [62]. These results indicate that a common early feature of WBR in these systems is the modulation of regulators of cell proliferation.

In addition to cell proliferation, these analyses suggest that cell death is tightly regulated early in regeneration. Genes associated with regulating cell death pathways are another example of similar differential expression early in these models. Notably, at least seven genes in the autophagy pathway are downregulated in regenerating sea star larvae, planaria, and hydra (i.e., *Atg16L1*, *Atg12*, *Atg10*, *Atg14*, and *Uvrag*; Fig. 6a). This is consistent with findings in hydra that suggest autophagic cell death is repressed during regeneration [63]. Conversely, as autophagy is downregulated in sea star larvae, genes that modulate apoptotic cell death are activated (e.g., *Fadd*, *Birc6*, and *Ulk1*). Apoptotic cell death is necessary for increased I-cell proliferation in hydra [18] and, in planarian regeneration, has been implicated in tissue remodeling and neoblast proliferation [64, 65]. Despite these early transcriptional changes, an increased number of TUNEL^+^ cells is not apparent until much later in bipinnaria regeneration (6 dpb; Fig. 4). Therefore, this modulation in cell death may be pathway specific (i.e., autophagy vs apoptosis) or otherwise undetected by our TUNEL assay. Alternatively, these transcriptional changes may be involved in establishing an appropriate balance between cell death and cell proliferation during this early phase.

Finally, we identified a suite of immediate early genes that are activated in all three animals. In regenerating sea star larvae, we find rapid, significant upregulation of *Jnk*, *Elk*, *Egr*, *Klf2/4, Mcl*, *Creb3l3*, *Fra2*, and *FoxO* (Fig. 6a). For example, *Egr* is one of the most strongly upregulated genes in both anterior and posterior regenerating sea stars (Fig. 6c), while in planarian regeneration *EGR* is one of the earliest and strongest wound-proximal genes induced during planarian regeneration [10]. The similar early downregulation of the *Egr* repressor *Toe1* in both sea stars and planaria suggests these genes are parts of concerted early response in these contexts. Several of these early activation factors are also known to be regulated by MAPK signaling pathways in other systems [66]. For example, in the sea urchin *Strongylocentrotus purpuratus*, *SpElk* is a target of MAPK signaling (ERK) and regulates both *SpRunt1* and *SpEgr* expression during embryogenesis [67]. In planaria, MAPK signaling (*Jnk*) activates *Runt1* and *Egr* following wounding [65]. *Jnk* signaling in hydra has been shown to regulate *FoxO* expression [68], which is an important regulator of hydra I-cells [69].

These overlapping sets of genes differentially expressed early reflect a common response to the bisection insult. This suggests that these gene orthologs define key shared characteristics between highly regenerative species in a specific response to injury that permits the regeneration program.

### Genes underlying shared early response are dramatically upregulated in the sea star wound site

We additionally chose a subset of these genes to examine their spatial localization during regeneration. *Elk* and *Egr* are both normally expressed in coelomic pouch epithelium (Fig. 6b″, c″), but by 3 hpb they are also strongly expressed in the sites of wound closure (Fig. 6b′, c′, Additional file 1: Figure S9 A, B). *Fgf9* expression is also localized to wound sites during early regeneration (Additional file 1: Figure S9 F). Although neither *Ets* nor *Erg* were significantly differentially expressed by RNA-Seq or nanostring, we examined their expression given their known expression in sea star mesenchyme [70]. We find that both are localized to wound sites during early regeneration (Additional file 1: Figure S9 D, E), suggesting an early role for mesenchymal cells, although not necessarily due to a transcriptional change. *Klf2/4* is normally expressed strongly in the mouth and foregut and after bisection is strongly upregulated in wound-proximal foregut (Fig. 6d′ and Additional file 1: Figure S9 C). Conversely, *FoxO*, *Jnk*, and *Runt* are expressed in the tip of the foregut proximal to the wound site, but not in the wound itself (Additional file 1: Figure S9 G–I). The tumor suppressor genes *Abl* and *Pten* are expressed broadly around the wound during early regeneration (Additional file 1: Figure S9 J, K). This spatial expression therefore shows that the set of gene homologs with early regenerative response among these deeply divergent animals are expressed in the early wound region of the sea star larva.

### Axis respecification precedes wound-proximal proliferation

Restoration of normal gene expression levels along the bisected AP axis must be a central component of regeneration. Gene expression domains for components of the GRN that controls early axial patterning in sea star embryo have been well defined. The Wnt pathway, for example, has well-characterized functions in specifying the embryonic AP axis [49, 70]. Anterior ectoderm domains required for the development of the larval nervous system have also been delineated [71–73]. This enables us to analyze the expression of these genes during regeneration. And indeed, analysis of genes within the two expression clusters differentially expressed in regenerating anterior and posterior larval fragments (clusters III and IV; Fig. 5) demonstrates that embryonic axis patterning genes are expressed during AP axis restoration.

**Fig. 7:**
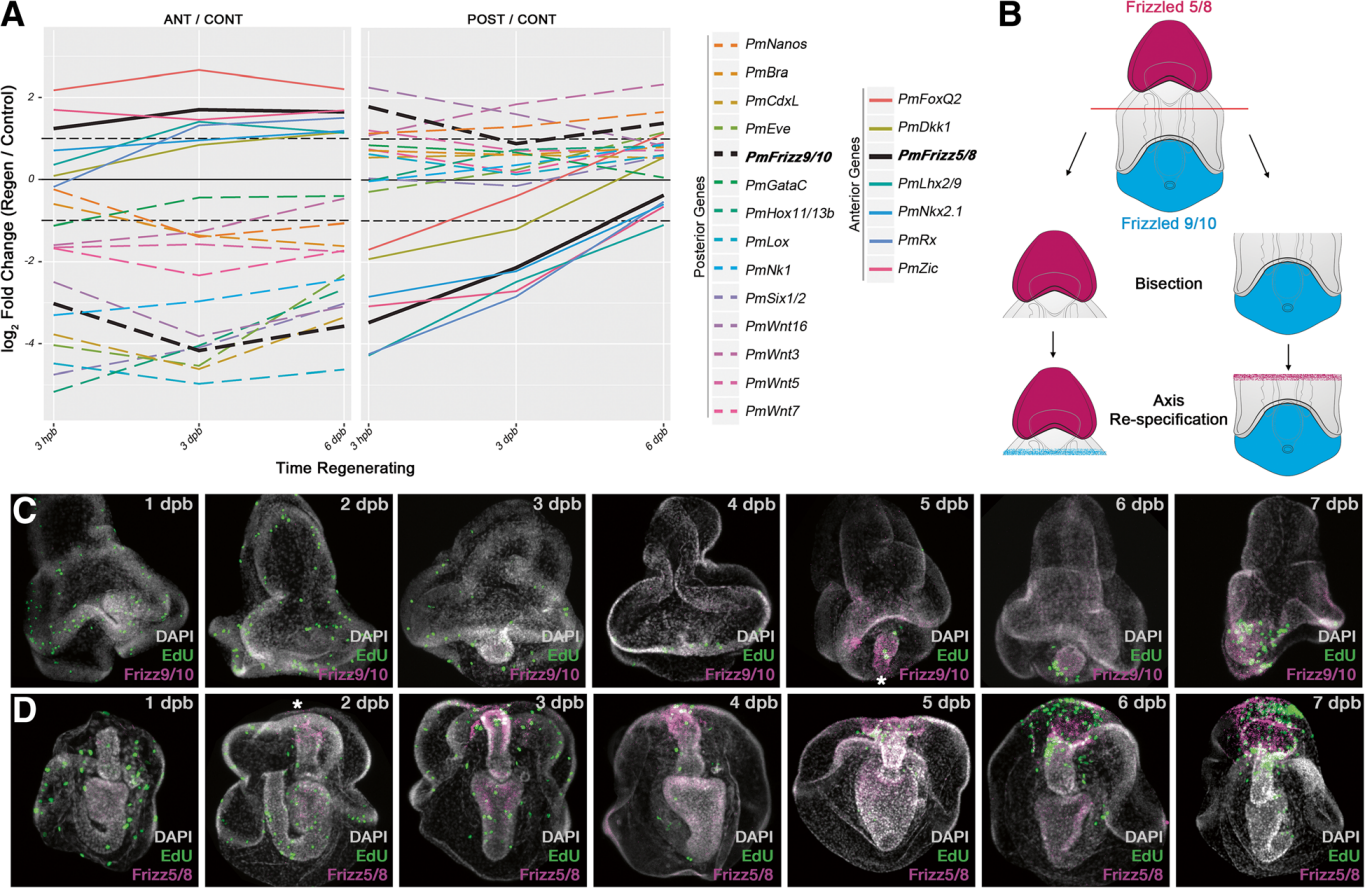
Fragment-specific recovery of appropriate anterior-posterior gene expression. **a** The expression of genes asymmetrically expressed in either anterior (ANT; solid lines, cluster III) or posterior (POST; dashed lines, cluster IV) sea star larval territories was examined at 3 h post-bisection (hpb), 3 days post-bisection (dpb), and 6 dpb. The log fold change values for each gene in regenerating anterior or posterior fragments compared with non-bisected sibling control larvae is reported for each fragment (ANT/CONT and POST/CONT, respectively) over the regenerating time course sampled. Black lines show the detected expression of *Frizz5/8* and *Frizz9/10*. **b** Model for recover of genes asymmetrically expressed along the anterior-posterior axis, with *Frizz9/10* (blue) and *Frizz5/8* (maroon) provided as examples. **c** Whole-mount fluorescent in situ hybridization illustrating the re-activation of *Frizz9/10* (magenta) in the posterior aspect of regenerating anterior fragments beginning at 5 dpb and preceding the concentration of proliferating EdU^+^ cells (green) near the wound site. **d** Re-activation of *Frizz5/8* (magenta) in the anterior aspect of regenerating posterior fragments beginning at 2 dpb and preceding the concentration of proliferating EdU^+^ cells near the wound site

When examining these clusters, it should be noted that although genes in these clusters appear to be rapidly downregulated following bisection, because transcript levels were normalized to those in whole larvae, this phenomenon is actually a result of removing cells and tissues in the other half of the larva. For example, genes normally expressed in anterior larval domains (e.g., *Frizz5/8* and *FoxQ2*) initially appear to be downregulated in posterior fragments relative to uncut larvae but are unaffected in anterior fragments (solid lines, Fig. 7; cluster III, Fig. 5). Correspondingly, genes that are typically expressed in the posterior domain (e.g., *Frizz9/10*, *Wnt16*, and *Nk1*) are absent in anterior fragments but unaffected in posterior fragments (dashed lines, Fig. 7; cluster IV, Fig. 5). For several genes in each of these clusters, expression levels recover to pre-bisection levels within 6 days. Notably, however, this process appears to be delayed within the regenerating anterior fragments relative to the posterior fragments (Fig. 7).

To characterize more fully the re-establishment of axial patterning during regeneration, we examined the spatial expression of two Wnt pathway receptor genes: *Frizz5/8* (normally expressed in the anterior) and *Frizz9/10* (localized in the posterior). In the anterior regenerating fragments, *Frizz9/10* transcripts are undetectable following bisection (immediately after the posterior halves were removed). However, by 5 dpb *Frizz9/10* transcripts are evident in the newly developed posterior domain (Fig. 7c). Additionally, we detect the re-expression of *Frizz9/10* before the onset of wound-proximal proliferation. Likewise, *Frizz5/8* is undetectable in regenerating posterior fragments until about 2 dpb when it is seen in the anterior aspect of these fragments (Fig. 7d), again before proliferating cells localize to this region. Appropriately localized expression of *Frizz9/10* and *Frizz5/8* persists in regenerating posterior and anterior fragments, respectively (Additional file 1: Figure S10 B, E). This finding extends to other genes with known roles in embryonic AP axial patterning that are identified in our clusters. For instance, we find similar recapitulation of embryonic expression patterns for, e.g., *FoxQ2* (another anterior marker) and *Wnt8* (an additional posterior marker; Additional file 1: Figure S10 F–J). Thus, embryonic patterning genes are used again during the restoration of the AP axis, and this precedes the initiation of blastemal proliferation.

This pattern mirrors planarian regeneration in which blastema formation, and regeneration cannot proceed when axis specification is perturbed [74–76]. Although hydra regeneration does not require blastemal proliferation, interstitial cells proliferate following wounding and this proliferation is initiated by a transient release of Wnt3, a protein implicated in head organizer function [18]. This comparison between animals positioned across the metazoa suggests the important finding that regeneration-associated proliferation requires a resetting of an axial positional program.

### Common regulatory toolkit used for axial respecification

We sought to determine if any of the genes involved in sea star axis respecification during regeneration are conserved among animals. We examined the genes assigned to these fragment-specific clusters (clusters III and IV) to identify orthologous genes with similar expression trends in the other datasets. We find significant overlaps between the posterior-specific sea star genes (cluster IV) and asymmetrically expressed genes in both hydra (cluster 1, Additional file 1: Figure S8) and planarian (cluster 2, Additional file 1: Figure S7) datasets. The hydra oral-aboral axis corresponds to the posterior-anterior axes in bilaterians [77]. The RNA-Seq data from hydra were generated using oral regions of the regenerating aboral body stalk [5]. Thus, the signature of late stage upregulation reflects the recovery of transcripts typically expressed in the head (cluster 1, Additional file 1: Figure S8) and we expect that oral gene expression in hydra would correspond to posterior gene expression in sea stars. These nominally oral-specific genes in hydra in fact do exhibit a significant overlap with the posterior-specific sea star genes (hypergeometric *p* = 2.7 × 10^−3^). Likewise, genes asymmetrically expressed between anterior and posterior halves in the planaria dataset overlap the posterior-specific sea star genes (hypergeometric *p* = 1.4 × 10^−2^). In both cases, the overlapping genes include Wnt ligands and receptors (e.g., *Wnt7*, *Wnt5*, and *Frizz9/10*) and other regulatory genes associated with posterior fates (e.g., *Bra*, *Hox11/13a*, and *Six1/2*). The observed overlap in asymmetrically expressed genes among these datasets suggests that a common regulatory toolkit is deployed for axis respecification in each of these models that includes Wnt signaling. The absolute orientation of the axes is not conserved, but this likely reflects developmental usage.

### Temporal dynamics of regeneration-induced cell proliferation differ among these animals

The patterns of cellular proliferation are one aspect in which the three models of WBR differ considerably. Sea star larvae and planaria exhibit concerted wound-proximal proliferation that coincides with the final time points sampled here: 6 dpb for sea star larvae and 3 dpb for planaria. Early in planarian regeneration, a global burst of neoblast proliferation is also observed (i.e., within 6 h post-amputation) [9]. No such early increase in proliferation is observed in sea star larvae (Fig. 3). While hydra do not rely on a proliferative blastema to resupply cells for regeneration, interstitial stem cells (I-cells) proliferate proximal to the wound within the first 2–4 h post-amputation [18]. This I-cell proliferation follows the early suppression of mitosis that is observed after wounding [5].

In sea star larvae, the genes upregulated later in regeneration in both the anterior and posterior fragments (cluster V; Fig. 5) are strongly associated with cell proliferation. It is important to note that while overall numbers of proliferation cells are decreasing, the timing of the upregulation of these genes correlates with the emergence of wound-localized proliferation. We compared these genes with orthologs that exhibit similar expression dynamics in the other datasets. None of the expression clusters from planaria or hydra are significantly enriched in orthologs of the sea star proliferation genes. Specifically, very few orthologs are apparent between the later upregulated sea star cluster (cluster V) and the corresponding gene clusters from planaria and hydra (i.e., planaria cluster 1 and hydra cluster 3; Additional file 1: Figure S7 and S8). Instead, there is a strong, though not statistically significant, overlap between the genes upregulated late in sea star and those upregulated early in planaria (e.g., cluster 3, Additional file 1: Figure S7) and hydra (e.g., cluster 1, Additional file 1: Figure S8). Many of these shared genes are associated with cycling cells (e.g., DNA polymerase subunits, MCM genes, structural maintenance of chromosomes [SMC] genes, *Orc3*, *Rrm1*, *Plk*, and *Ttk*). These data suggest the intriguing hypothesis that wound-proximal proliferation in sea star larvae is more similar to early bursts of cell proliferation than the later blastemal proliferation observed in planarian regeneration.

### Regeneration induces coincident expression of normally tissue restricted proliferation-associated genes

**Fig. 8:**
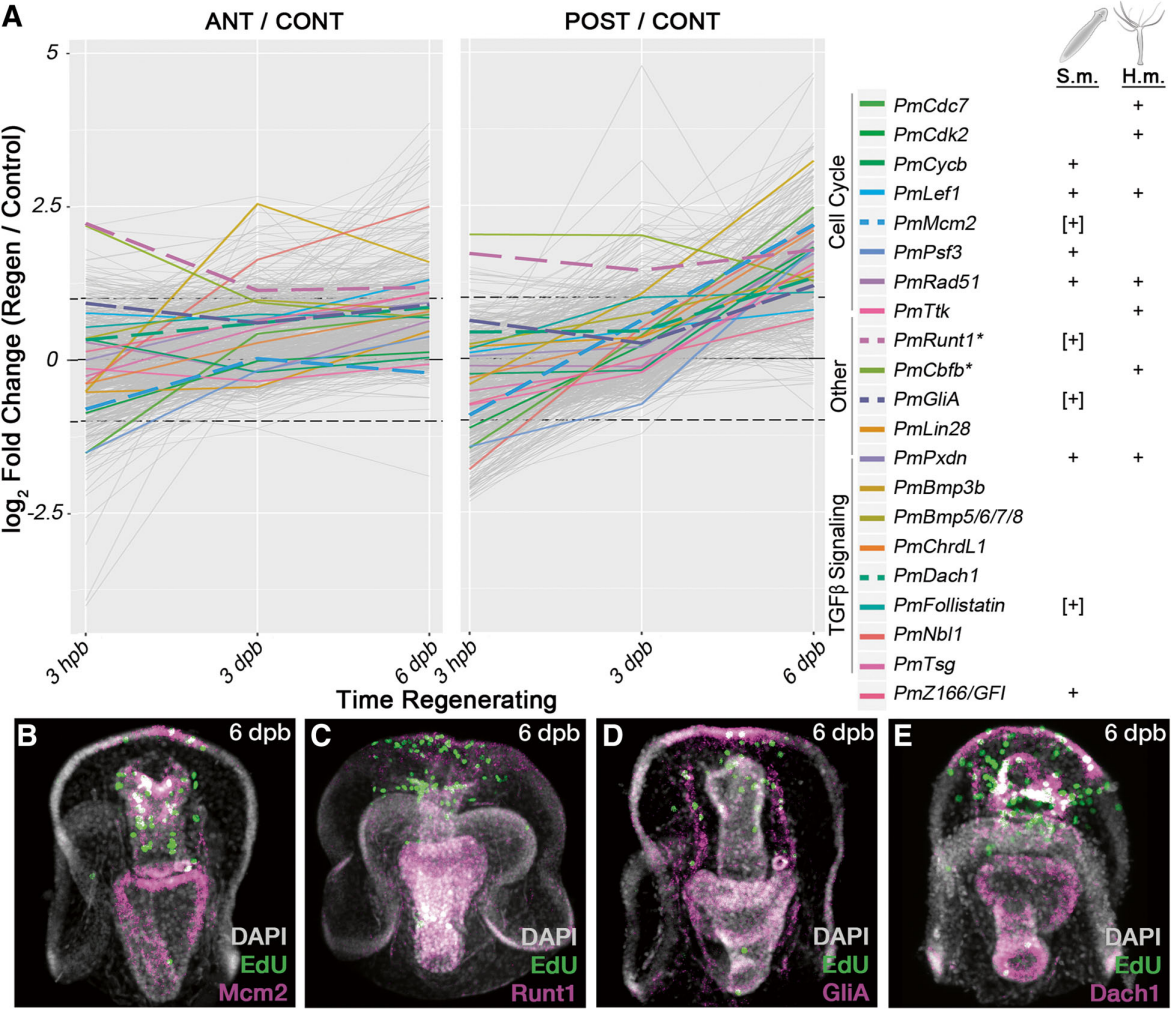
Shared proliferation-associated genes. **a** These data show sea star log fold change values for genes differentially expressed at later stages in regenerating fragments compared with non-bisected sibling control larvae (i.e., sea star cluster V). All genes assigned to cluster V are plotted in gray. Several genes, either referenced in the text or representative of functions considered, are indicated with colored lines. Next to the key for each gene is an indication (i.e., “+”) of whether an ortholog for that gene was found in an analogous cluster in either the planaria (*S.m.*) or hydra (*H.m.*) datasets. Indicators in brackets (e.g., “[+]”) are those where no overlapping ortholog was identified by our analyses, but genes with the same name were implicated by published datasets. Genes plotted with dashed lines are shown by fluorescent in situ hybridization (below). *Mcm2* (**b**), *Runt1* (**c**), *GliA* (**d**), and *Dach1* (**e**) are all expressed in the anterior aspects of regenerating fragments at 6 dpb. In many cases, the expression of these genes is coincident with an EdU^+^ cell, suggesting that these genes are expressed, at least in part, in proliferating cells

We examined the expression patterns of proliferation-associated genes during regeneration (i.e., cluster V). *Mcm2*, *Runt1*, *GliA*, and *Dach* are all expressed in the anterior region of regenerating posterior fragments, coincident with the wound-proximal proliferation (Fig. 8b–e). Each gene is expressed in multiple distinct tissues, including the anterior foregut, anterior epithelium, coelomic epithelium, and gut (Fig. 8b–e). Notably, however, during embryonic and larval stages, these genes exhibit non-overlapping expression patterns. For example, *Mcm2* is expressed in the ciliary band and foregut; *Runt1* is expressed in the mouth, midgut, and hindgut; *GliA* is strongly associated with the developing coelomic epithelium; and *Dach* is expressed throughout the gut and in ciliary band epithelium (Additional file 1: Figure S11). These results indicate that a suite of genes that function in cell proliferation and are normally expressed in diverse tissues are re-deployed during regeneration and are co-expressed in the proliferating blastema.

## Conclusion

While the capacity for larval sea stars to undergo WBR has been appreciated for over two decades, there has not yet been a systematic characterization of the cellular and molecular processes involved. In the present study, we demonstrate that larval sea stars exhibit many stereotypical characteristics found in other models of WBR. This is a striking finding because sea stars are Deuterostome animals and very distantly related to the other species considered here. Through our transcriptome analyses, we detect an early wound-response phase involving significant alterations in the expression of stress response genes, genes involved in signaling pathways (including MAPK, Ca^2+^) and a broad shut-down of energetically expensive anabolic processes (e.g., ribosome biogenesis). The first few days following bisection are marked by a global decrease in the number and distribution of cycling cells compared to what is typically observed in growing, non-bisected larvae. This precedes the re-establishment of developmental axes, specifically the AP axis ablated by bisection. Re-patterning of the AP axis is observed both through in situ hybridization as well as transcriptome measurements. These observations are facilitated by our extensive prior knowledge of sea star developmental patterning programs, and, indeed, genes described by the developmental gene regulatory network are enriched in these clusters. Notably, through both our transcriptome and in situ experiments, we observe that axis respecification occurs prior to the onset of wound-proximal cell proliferation, which is the last phase assayed in the present study. This is the first description of concerted, wound-proximal cell proliferation in regenerating sea star larvae. Given that this wound-facing region in both regenerating fragments is the primordium from which larval tissues regenerate, we define this proliferative zone as the regeneration blastema. In this study, we have only monitored the first half of the regeneration process up until the emergence of this blastema. Complete regeneration in these larvae takes a total of 10–14 days [42, 43].

**Fig. 9:**
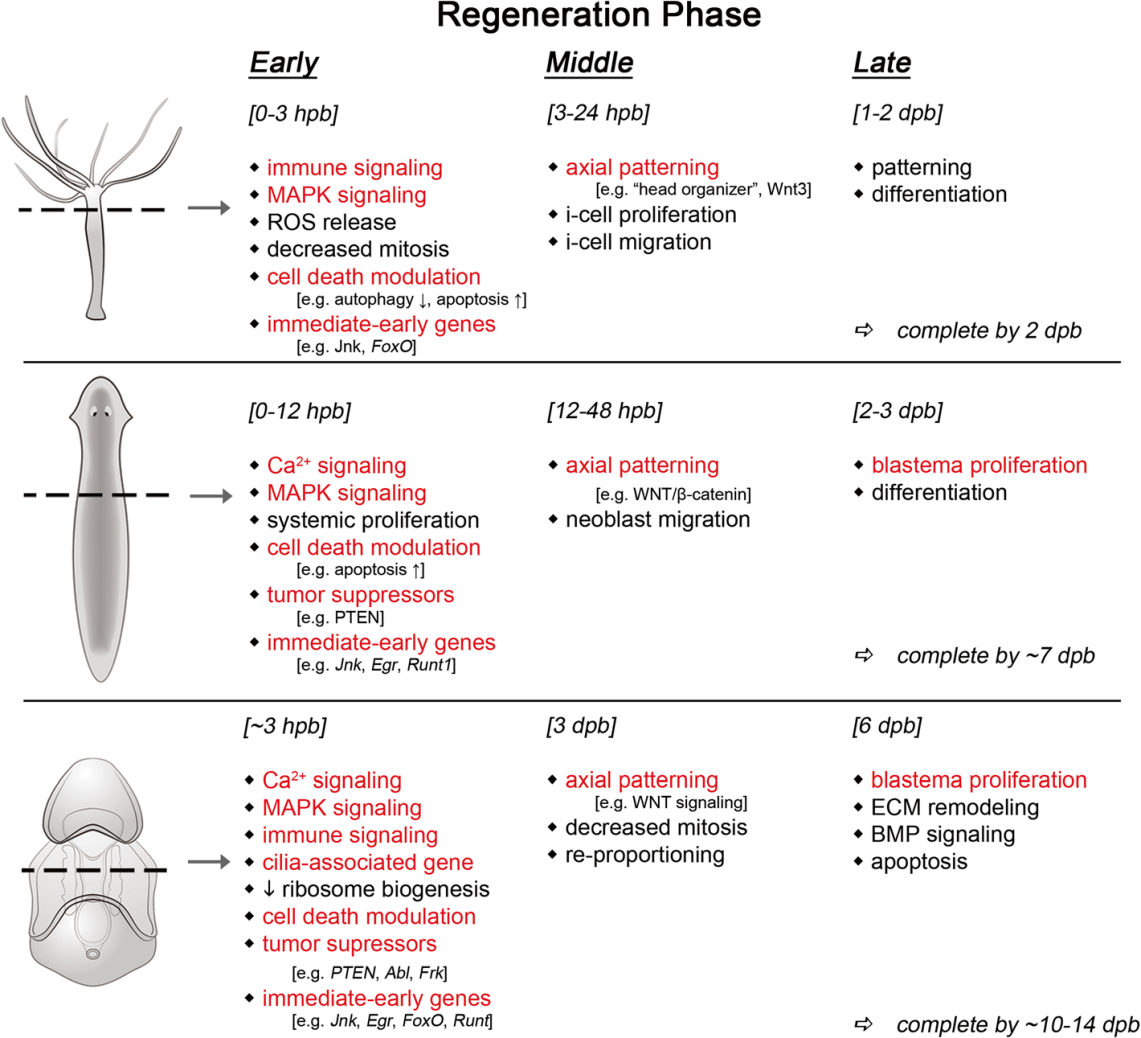
Summary of similarities between WBR models. The reported features of regeneration at early, middle, and late stages of regeneration, with respect to the datasets considered in this study, are indicated. Features detected in the sea star model in our study that are shared with the other two models are highlighted in red. Some aspects are considered in common based on shared gene expression (e.g., MAPK signaling) whereas others are based on cytological observations (e.g., blastema proliferation)

In this work, we sought to leverage the power of comparing regeneration in a variety of contexts to identify common features. For example, we clustered gene expression levels to identify genes similarly differentially expressed in both anterior and posterior regenerating sea star larval fragments. These patterns were then used as a basis for comparison to planaria and hydra regeneration datasets. In the present study, we compared regeneration in species that last shared a common ancestor approximately 580 million years ago, at the base of the metazoa. This is the broadest direct comparison of regeneration yet described, encompassing three of the major groupings of animals (Deuterostome, Protostome, and basally branching Eumetazoa). We find evidence for similarities in the use of both broad functional classes as well as specific orthologs involved with the regenerative process among these animals. Such similarity can imply conservation—i.e., that these genes and processes are homologous and maintained from a common ancestor—or could suggest independent co-option into distinct regenerative processes. Indeed, the genes in common are orthologs with deeply conserved functions in core cellular processes that are required in many regenerative contexts (e.g., cellular proliferation and apoptosis). The significance of our finding here is not that we detect such genes, but that we find evidence for shared temporal expression in many of these processes. Furthermore there are also examples of genes with divergent expression patterns among these animals. In this work, we focus our attention on those that are shared as these have the greatest potential to inform our goal of identifying common features of highly potent regeneration. As with any EvoDevo study, it is difficult to absolutely distinguish between a genuine homology of these regenerative programs, rather than independent convergence of multiple critical pathways. These commonalities are summarized in Fig. 9. The most remarkable signature of shared genes and processes is among genes both up- and downregulated early. We are potentially most empowered to detect such an overlap among early genes as temporal synchrony between the models likely diverges later in the time course. Nonetheless, early changes to Ca^2+^ and MAPK signaling pathways, upregulation of ciliogenesis genes, upregulation of tumor suppressor genes, downregulation of autophagy genes, and activation of a suite of immediate early genes are common aspects of regeneration in these models. There is also a set of similarly expressed genes that we hypothesize are commonly involved in axial respecification, most notably genes in the WNT signaling pathway. Importantly, axis respecification occurs prior to regeneration-associated proliferation across these species. In contrast to these commonalities, we show that the temporal profiles of gene expression underlying the proliferative response are different.

These commonalities between highly diverged WBR models highlight a deep similarity in regeneration mechanisms among the metazoa. This work also underscores the power of comparative inquiries in identifying the core components of the regenerative response and, potentially, how these components are altered in non-regenerative species.

## Methods

### Animals and regeneration paradigm

Adult *Patiria miniata* were obtained from the southern coast of California, USA (Pete Halmay or Marinus Scientific) and were used to initiate embryo cultures as previously described [78]. *P. miniata* embryos were cultured in artificial seawater at 16 °C and fed *Rhodomonas lens* ad libitum every 2 days along with fresh artificial seawater beginning at 4 days post-fertilization (dpf). All studies of regenerating larvae were conducted with larval cultures beginning at 7 dpf at which point the larvae were manually bisected stereotypically through the foregut, midway along the transverse anterior-posterior axis (Fig. 1b), with a #11 sterile scalpel. Resulting anterior and posterior fragments, as well as control (uncut) larvae, were then transferred to separate 35-mm polystyrene dishes at a density of no more than 50 larval fragments per milliliter of artificial seawater and cultured for the time indicated.

### Whole-mount staining and staining larval sections procedures

*P. miniata* larvae or regenerating larval fragments, grown for the times indicated, were fixed in a solution of 4% paraformaldehyde in MOPS-fix buffer (0.1 M MOPS pH 7.5, 2 mM MgSO_4_, 1 mM EGTA, and 800 mM NaCl) for 90 min at 25 °C and transferred to a solution of 70% ethanol for long term storage at − 20 °C. In situ hybridization experiments were performed as previously described [71, 79] using digoxigenin-labeled antisense RNA probes. Labelling and detection of proliferating cells in *P. miniata* larvae were performed using the Click-it Plus EdU 488 Imaging Kit (Life Technologies), with the following modifications. Larvae were incubated in a 10 μM solution of EdU for 6 h immediately prior to fixation in a solution of 4% paraformaldehyde in phosphate buffered saline (PBS). Larvae were fixed for a minimum of 90 min at 25 °C and subsequently transferred to a solution of 70% ethanol for storage at − 20 °C. For detection of EdU incorporation, labeled embryos were transferred to a solution of PBS and the detection was performed following the manufacturer’s protocol.

For detection of in situ and EdU staining in the same specimen, EdU-labeled larvae were fixed and hybridized with digoxigenin-labeled riboprobes, as described. Detection was performed using a 1:1000 dilution of anti-digoxigenin POD-conjugate antibody (Roche Cat# 11207733910, RRID:AB_514500) and tyramide signal amplification (Perkin Elmer). Following signal deposition, larvae were washed in PBS and EdU was detected as described.

For BrdU pulse-chase experiments, larvae were labeled with 50 μg/ml solution of BrdU (Sigma B5002) for 6 h after which they were washed and placed in fresh seawater. Following fixation, larvae were denatured in 2 M HCl and 200 mM NaCl for 30 min at 37 °C. The denaturant was neutralized in 0.1 M Borate buffer (pH 8.5), followed by blocking in PBS with 2% BSA and 0.1% Tween 20. The anti-BrdU antibody (Sigma-Aldrich Cat# B2531, RRID:AB_476793) was diluted 1:100 in blocking buffer incubated for 1 h. The larvae were then washed in PBS with 0.5% Tween-20 and incubated in a 1:500 dilution of anti-mouse Alexa 568 (Thermo Fisher Scientific Cat# A-21124, RRID:AB_2535766) for 1 h. Following additional PBS washes, EdU detection was performed as described.

For TUNEL staining, animals were fixed in 4% paraformaldehyde in 0.01 M phosphate buffer (pH 7.4, 1007 mOsm) for 24 h at 4 °C. After fixation, the embryos were incubated in 0.1 M glycine in phosphate buffer with 0.1% Tween 20 for 1 h to quench residual autofluorescence. The tissues were permeabilized in 0.5% Triton X-100 for 30 min and by Proteinase K digestion (8 μg/ml, 10 min at room temperature). Cells undergoing programmed cell death were identified using the Fluorescent FragEL™ DNA Fragmentation Detection Kit (Calbiochem) as per the manufacturer’s protocol. Images of whole-mount specimens were taken using the Zeiss LSM 880 scanning laser confocal microscope. Maximum intensity Z-projections and automatic cell counting were generated in the Fiji image processing software.

At least two independent biological replicate experiments were performed for each in situ, EdU staining, or TUNEL staining experiment, examining the pattern of at least 10 specimens per replicate. For quantitation of EdU and TUNEL images, Z-projections were generated and counted in ImageJ. Images were converted to 16-bit prior to thresholding. For images of anterior larval segments, a 0.4% threshold was used, and for images of uncut larvae and posterior segments, a 1% threshold was used. Each image was then converted to a binary mask shed. Using the Watershed tool, larger objects were segmented into individual cells. To segment each image into three sections (wound, middle, distal), each image was divided into three equal portions. To quantify the number of EdU+ cells in each section, the Analyze Particles tool was used. For uncut larvae, the size parameters used was 5–300 μm^2^ and for regenerating larvae, 20–300 μm^2^. Statistical analysis of count data was performed using the estimation stats website [80] and, in all cases, used the 0 dpb as a shared control sample, and reported *p* values are based on nonparametric Mann-Whitney *U* tests.

For histology, larvae were fixed in 4% paraformaldehyde in 0.01 M phosphate buffer (pH 7.4, 1007 mOsm). After fixation, the specimens were rinsed in the same buffer and postfixed in 1% OsO_4_ for 1 h. The samples were dehydrated in a graded series of ethanol and propylene oxide and embedded in the Araldite epoxy resin. Sections were cut with glass knives on Ultracut E (Reichert, Vienna, Austria). The serial semi-thin (1 μm) sections were collected on gelatin-coated slides, stained with 1% toluidine blue in 1% aqueous sodium borate and mounted in DPX (Fluka). The sections were viewed and photographed with a Leica DMI 4000B microscope equipped with a Leica DFC 420C camera.

### RNA-Seq, read mapping, and transcriptome assembly

For transcriptome measurements, larvae were grown and bisected as described in the results. RNA was collected from pools of approximately 300 sibling individuals of regenerating anterior fragments, regenerating posterior fragments, as well as uncut control larvae. Two biological replicate samples were prepared for each timepoint for a total of 18 samples. RNA was extracted using the GenElute Mammalian Total RNA Kit (Sigma-Aldrich). Illumina TruSeq library preparation and HiSeq 2500 50 bp SR sequencing were performed (USC Epigenome Center).

RNA-Seq reads were trimmed of residual adapter sequences and low-quality bases (Trimmomatic v0.32 [81]). High-quality reads were mapped to the *P. miniata* v1.0 genome assembly (Tophat v2.0.12 [82]), and in total, 422.9 M uniquely mapping reads were recovered from the 18 samples at an average depth of 23.5 M reads per sample. Uniquely mapping reads were assembled into transcripts using Cufflinks [83], and the MAKER2-based gene predictions hosted at Echinobase were used to guide transcript assembly. Reads uniquely mapping to a gene (locus) from this Cufflinks transcriptome assembly were counted (HTSeq-count v0.6.1p1 [84]). Read counts were normalized, and genes detected with more than three reads per million, corresponding to 50–120 uniquely mapping reads depending on the sample, in at least two samples were retained for further analyses, corresponding to 31,798 expressed genes. Raw and processed sequencing reads have been deposited into the NCBI Gene Expression Omnibus (GSE97230) [85] and analysis scripts are available upon request.

### Gene Ontology term annotation and ortholog identification

The newly assembled sea star genes were annotated in three ways: by identifying the reciprocal best BLAST hit (rBBH) between the sea star transcript and either sea urchin or mouse genes and using Blast2GO. Nine thousand twenty-seven (28.4%) loci have an rBBH match to a sea urchin protein, 7212 (22.7%) loci have an rBBH match to a mouse gene, and 9617 (30.2%) assembled loci were annotated using Blast2GO. GO terms for each sea urchin and mouse genes were assigned to their respective rBBH match in the sea star set, and these were used for enrichment analyses. Overall, the results based on all three annotation methods are highly similar (Fig. 3b and Additional file 1: Figure S6). Reciprocal best BLAST hits (rBBH) were also used to identify putative orthologs between the sea star genes and the planaria and hydra transcripts. We found 5220 *S. mediterranea* transcripts and 6091 *H. magnipapillata* transcripts with an rBBH match to a sea star transcript. The identified orthologs for each sea star transcript are reported (Additional file 2: Table S1).

### Differential expression testing and hierarchical clustering

Expression levels in biological replicate samples are highly correlated (Pearson correlation coefficient = 0.985). Regenerating fragments were compared to age-matched sibling uncut control larvae and differential expression was assessed using a generalized linear model quasi-likelihood F test (edgeR [86, 87]), controlling for sample batch. Differentially expressed genes (DEG) were defined as those changes detected below a *p* value of 0.05 and with a fold change greater than twofold in either direction. Using these criteria, there are 9211 total DEG in at least one regenerating fragment compared to the control larvae and at least one of the timepoints sampled, which represents 28.97% of all of the expressed genes detected (Additional file 2: Table S1).

The fold change values for all 9211 DEG relative to control larvae were clustered by first computing the euclidean distance matrix, and then, these values were then clustered using the “ward.D2” method provided as part of the R hclust function. The optimum number of clusters was determined by cutting the resultant dendrogram at various heights and empirically determining at which height the number of clusters began to plateau (*h* = 42). The result was eight distinct clusters. However, we noted that several clusters shared similar overall patterns (Additional file 1: Figure S4). As the similar clusters shared very similar GO enrichments and expression patterns over the time course, we further grouped these into the final five clusters reported in the text. The grouping of clusters did not alter the enrichment of GO terms or our other downstream analyses (Additional file 1: Figure S6).

For the planaria and hydra regeneration datasets, data was obtained from supplemental tables associated with each publication. The planarian data were reported as normalized read counts for the 15,422 transcripts detected. These counts were log_2_-transformed and then scaled to *z*-scores, or the number of standard deviations from the mean value for each transcript, and only those transcripts considered differentially expressed as reported by the authors were considered. This resulted in 7975 transcripts that were then clustered in the same way as described above for the sea star transcripts. The hydra data were reported as binned *z*-scores for the 28,138 transcripts detected corresponding to lower, mid, and upper third of expression range for each transcript. We only clustered transcript values for which a positive reciprocal match was detected, leaving 5779 transcripts for our analyses. The euclidean distance matrix was calculated, as with the other datasets, but to accommodate the binned nature of these data the hierarchical clustering was performed using the “average” method provided with the hclust R function. A fine-grained resolution of common gene expression dynamics across these species is not warranted without more closely aligning experimental designs, including sampling time points and normalization strategies. Therefore, for each of these datasets, we sought very broad cluster classifications such that assigned genes are either upregulated early and down later or vice versa in their respective time course. The result is three clusters each for the *S. mediterranea* and *H. magnipapillata* datasets (Additional file 1: Figure S7 and S8).

### Nanostring nCounter assay analysis

A custom Nanostring nCounter codeset was designed, available upon request, consisting of 114 total probes—8 negative control, 6 postitive control, 11 housekeeping control, and 89 gene-of-interest probes. RNA was prepared from similarly staged larvae and hybridized to the codeset as directed by the manufacturer. The nCounter DA71 digital analyzer output files were collected, and further analysis was performed using the NanoStringDiff R package [88]. Briefly, background signal was defined for each sample as the mean plus two standard deviations of the negative control probes and assigned as the negative control normalization factor parameter. The geometric mean of signals for each sample from positive control probes and housekeeping probes were used to calculate a positive control and housekeeping scaling vectors for each sample. Differential expression between regenerating fragments and control, uncut larvae was determined using a generalized linear model likelihood ratio test (*p* < 0.05). Probes that failed to express above background levels were omitted from further analyses (Additional file 3: Table S2). Finally, heatmaps of fold change calculated based on Nanostring measurements were plotted for genes assigned to groups based on RNA-Seq cluster identities (Additional file 1: Figure S5). Genes with similar general expression dynamics (e.g., up early in both fragments, down early in both fragments, etc) in both RNA-Seq and Nanostring experiments were detected.

## Additional files

Additional file 1: **Figure S1.** Anterior fragment regeneration. **Figure S2.** Data used to make length ratio measurement. **Figure S3.** TUNEL signals are unchanged at 3 hpb and do not localize through 6 dpb. **Figure S4.** Sea star cluster consolidation. **Figure S5.** Nanostring nCounter validation of RNA-Seq data. **Figure S6.** Gene ontology enrichments. **Figure S7.** Comparison of *P. miniata* and *S. mediterranea* datasets. **Figure S8.** Comparison of *P. miniata* and *H. magnipapillata* datasets. **Figure S9.** Additional genes that are upregulated early and localized to the wound site. **Figure S10.** Recovery of anterior-posterior axis specification genes. **Figure S11.** Whole mount in situ hybridization showing larval and embryonic expression of genes associated with proliferation. (PDF 1936 kb)

Additional file 2: **Table S1.** RNA-Seq results. Table summarizing results of *P. minata* RNA-Seq experiments including each identified gene (“xloc”) along with a human-readable gene name and corresponding PMI accession based on Echinobase Pmin v1.0 annotations. For each gene, it is indicated whether it was found to be differentially expressed in any sample (“DE”) along with the fold change values for anterior and posterior regenerating fragments, relative to controls, for 0, 3, and 6 dpb. In addition, for each gene, if an ortholog was identified by reciprocal best blast in *S. purpuratus*, *S. mediterranea*, *H. magnipapillata*, or *M. musculus*, the accession of the identified ortholog is indicated. (CSV 4973 kb)

Additional file 3: **Table S2.** Nanostring nCounter results. Table summarizing the results of Nanostring nCounter experiments used to validate the RNA-Seq dataset. The table consists of the probe name as well as the pertinent identifiers, including xloc and tcons and a binary T/F flag indicating whether the probe was determined to be working. The observed fold change in expression from anterior and posterior fragments relative to controls at 0, 3, and 6 dpb are shown (e.g., a0_c0, a3_c3, etc). Finally the sequence of each probe is indicated along with the Nanostring probe accession. (CSV 23 kb)

## Abbreviations

ANT: Anterior
AP: Anterior-posterior
CONT: Control
DEG: Differentially expressed gene
dpb: Days post-bisection
GO: Gene Ontology
GRN: Gene regulatory network
*H.m.*: *H. magnipapillata*
hpb: Hours post-bisection
POST: Posterior
rBBH: Reciprocal best blast hit
*S.m.*: *S. mediterranea*
WBR: Whole-body regeneration
