# Supplementary material for: Analysis of sea star larval regeneration reveals conserved processes of whole-body regeneration across the metazoa
Source: BMC Biol. 2019 Feb 22;17:16. doi: 10.1186/s12915-019-0633-9 (PMC6385403; doi:10.1186/s12915-019-0633-9)
Supplement: Supplementary file 1 — Figure S1. Anterior fragment regeneration. Figure S2. Data used to make length ratio measurement. Figure S3. TUNEL signals are unchanged at 3 hpb and do not localize through 6 dpb. Figure S4. Sea star cluster consolidation. Figure S5. Nanostring nCounter validation of RNA-Seq data. Figure S6. Gene ontology enrichments. Figure S7. Comparison of P. miniata and S. mediterranea datasets. Figure S8. Comparison of P. miniata and H. magnipapillata datasets. Figure S9. Additional genes that are upregulated early and localized to the wound site. Figure S10. Recovery of anterior-posterior axis specification genes. Figure S11. Whole mount in situ hybridization showing larval and embryonic expression of genes associated with proliferation. (PDF 1936 kb) [file 12915_2019_633_MOESM1_ESM.pdf]

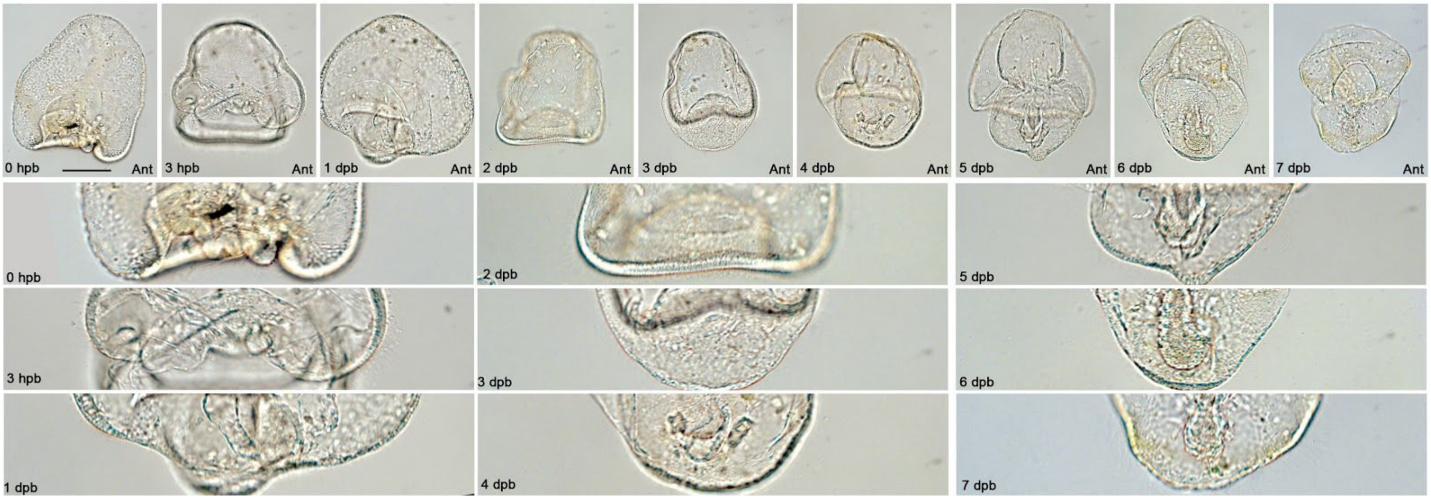

Figure S1. Anterior fragment regeneration. Regeneration of anterior fragments shows many of the same features as posterior fragment regeneration including wound closure and healing between 3 hours post bisection (hpb) and 1 day post bisection (dpb). We note that by 6-7 dpb there appears to be a rudimentary gut within the posterior aspect of anterior fragments, suggesting a re-specification of larval tissues by this time. Scale bar = 100 μm; applicable to all images.

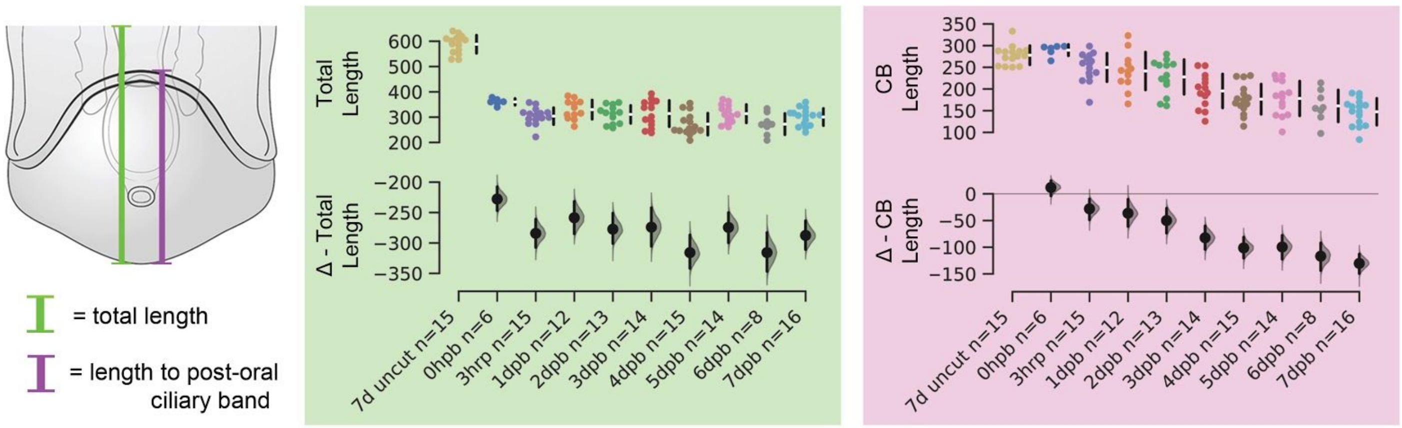

Figure S2. Data used to make length ratio measurement. Total length (green box) is decreased relative to control larvae immediately upon bisection and remains low past 7 days post bisection. The distance from the posterior pole to the top of the post-oral ciliary band (pink box) is relatively unchanged early, but gradually diminishes over the time course. Each metric is plotted along with the difference of the means (i.e. Δ Total Length and Δ CB Length) and 95% confidence interval. n=number of individuals measured for each timepoint.

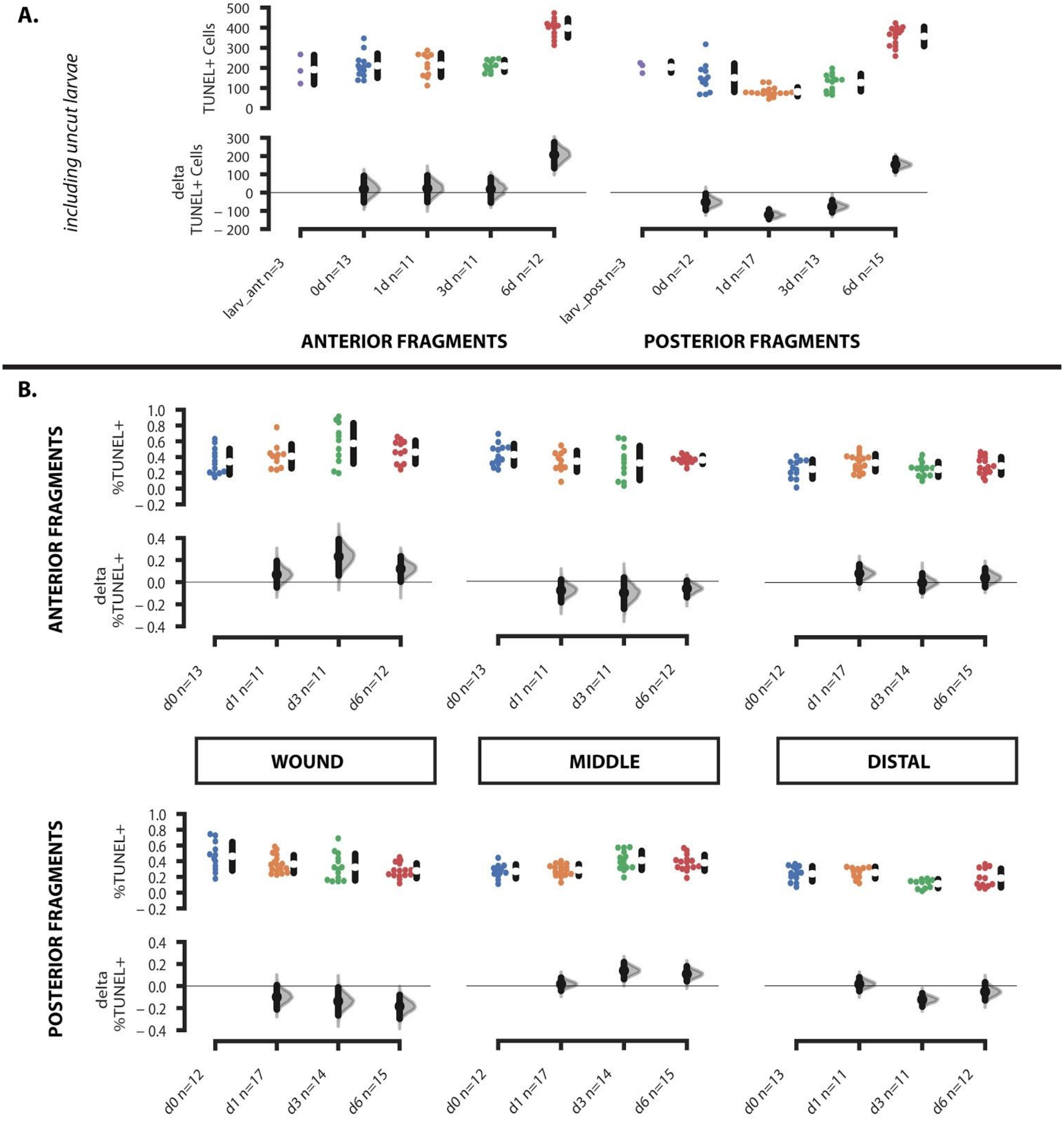

Figure S3. TUNEL signals are unchanged at 3 hpb and do not localize through 6 dpb. (A) The count of TUNEL⁺ cells, including counts in anterior and posterior halves of non-bisected larvae, are indicated to demonstrate that there is no increase relative to the level of apoptosis in normal larvae. (B) The fraction of TUNEL⁺ cells in wound-proximal, middle, and distal thirds of each regenerating fragment are shown. For each comparison a difference of the mean (i.e. Δ % TUNEL⁺) shown. No significant differences are detected.

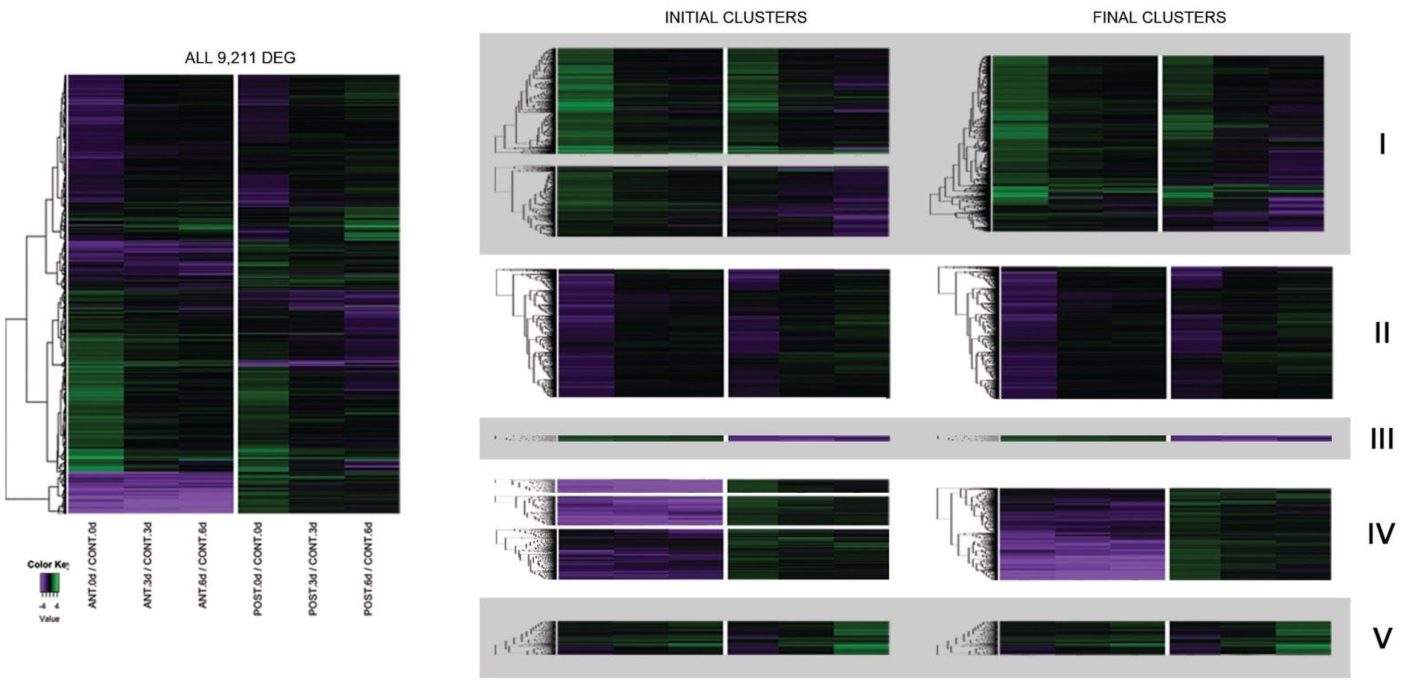

Figure S4. Sea star cluster consolidation. The dendrogram resulting from the hierarchical clustering of 9,211 DEG (left) was cut to yield eight clusters (middle, “initial clusters”). Green indicates a positive fold change (upregulated with respect to uncut controls), whereas purple indicates a negative fold change (down-regulated with respect to control).These were then grouped as indicated, based on similar trends, to yield the final five clusters (right).

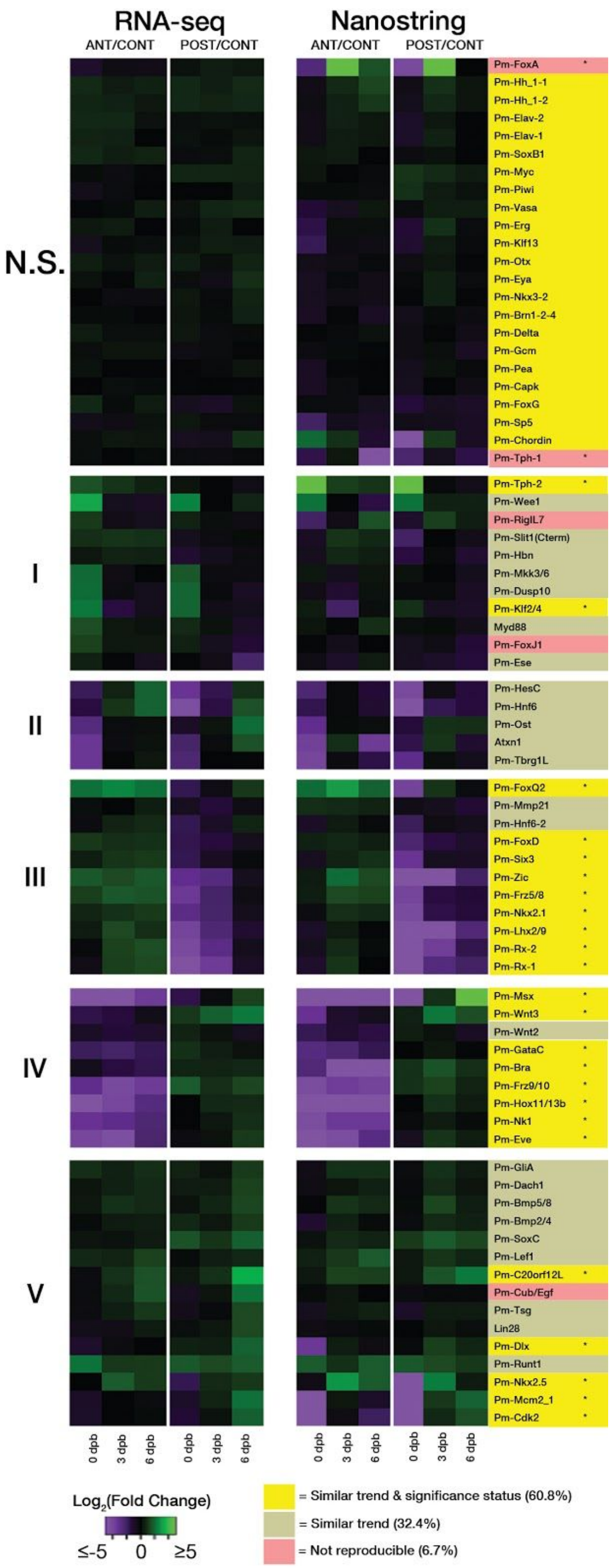

Figure S5. Nanostring nCounter validation of RNA-seq data. Heatmaps show the measured fold-change between regenerant and control samples from RNA-seq (left) and Nanostring (right) experiments for the genes analyzed using the Nanostring nCounter codeset. Clusters are assigned based on RNA-seq expression profiles (Fig 5, i.e. I-V as well as not significant N.S.). Each gene that was significantly differentially expressed in any of the Nanostring comparisons ($p < 0.05$) are indicated with an asterisk. Finally genes are grouped into similar significance finding (i.e. not significantly differentially expressed in both, or significantly differentially expressed in both) and similar expression trends (i.e. up early, up late, etc).

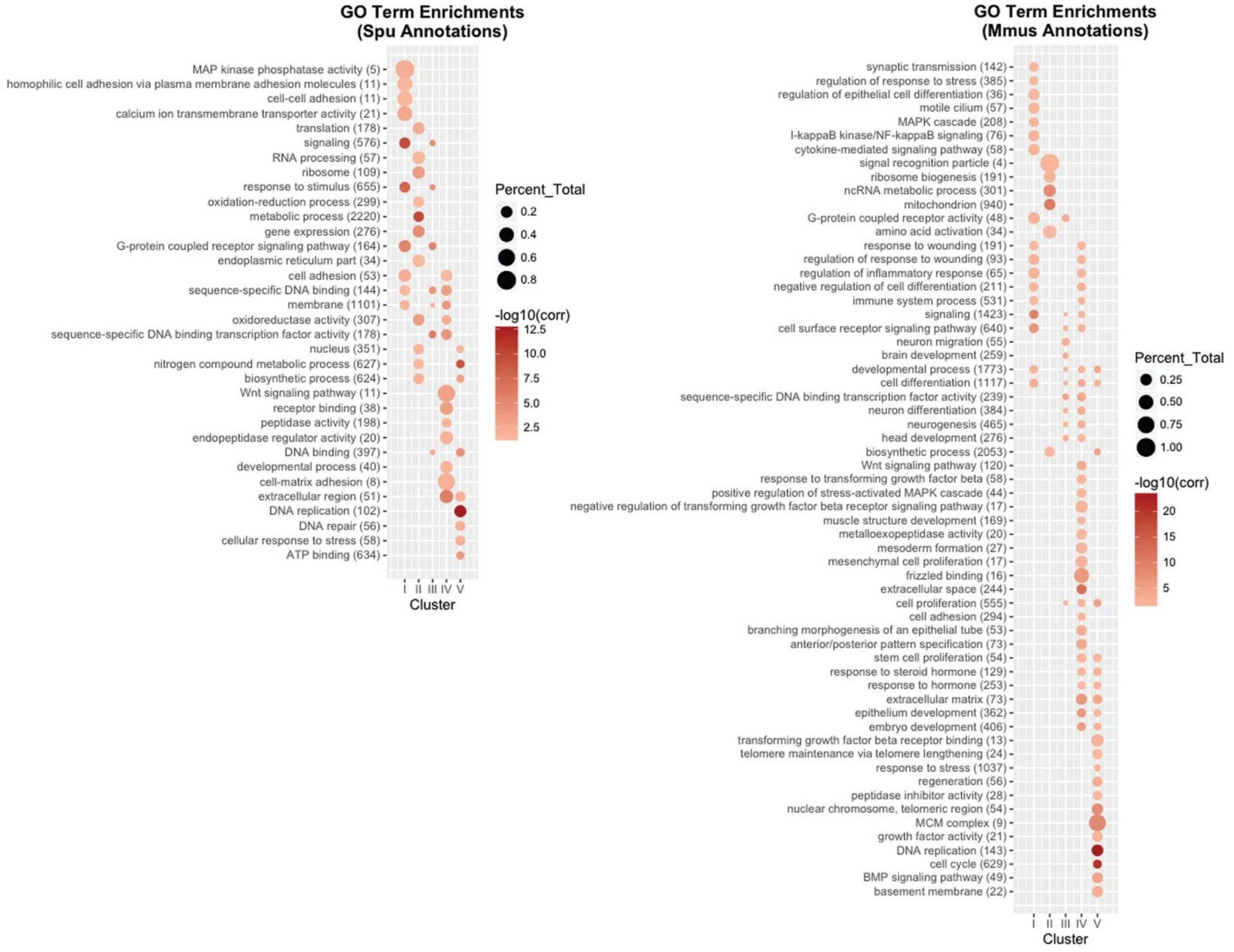

Figure S6. Gene ontology enrichments. Gene ontology enrichment analysis based on annotations from identified sea urchin, *Strongylocentrotus purpuratus* (left), and mouse, *Mus musculus* (right), orthologs for each RNA-seq cluster. The enrichment of each GO term is indicated by a circle where the area corresponds to the fraction of genes annotated with that term are present in the cluster, and the color of the circle corresponds to the corrected hypergeometric p-value of term enrichment.

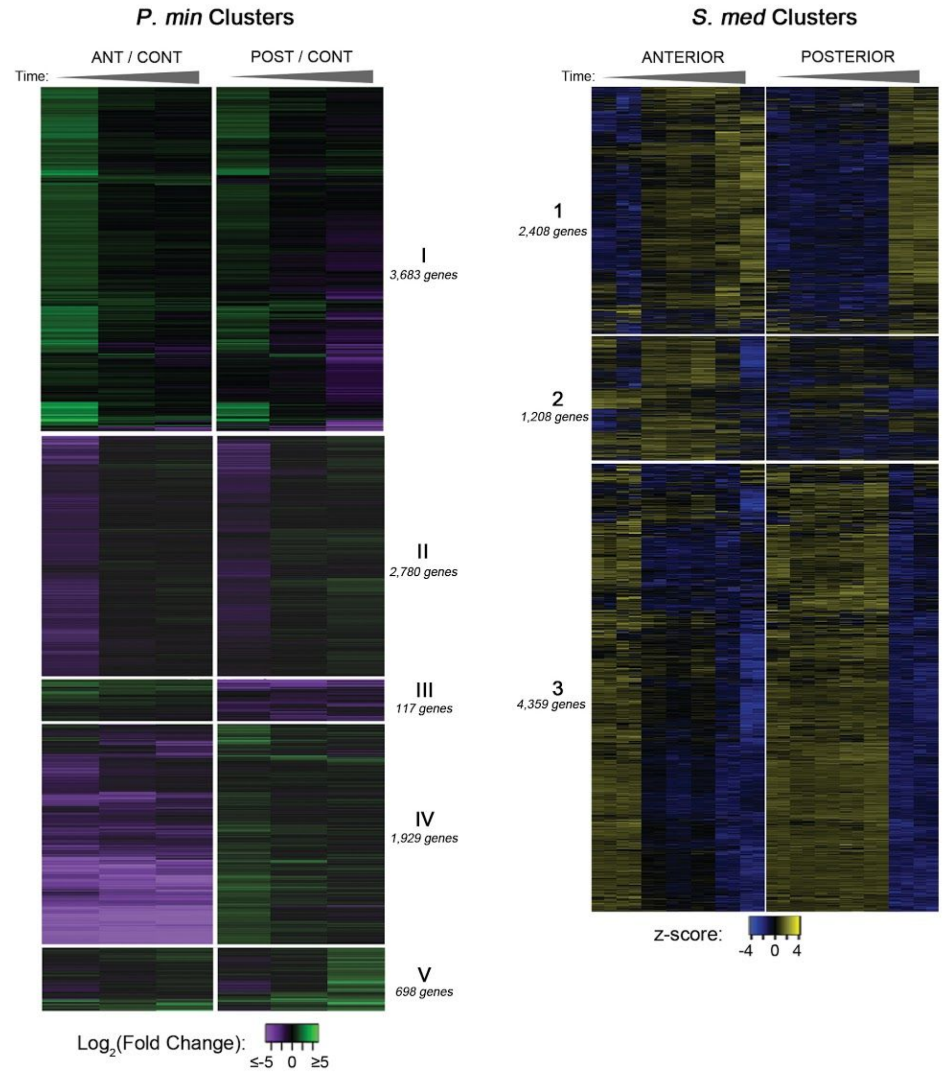

Number of Overlapping Orthologs

P. min Clusters (columns); *S. med* Clusters (rows)

	I	II	III	IV	V
1	38	136	0	18	14
2	48	48	2	24	8
3	147	90	2	43	40
	658 total orthologs				

Scale: *Number Overlapping*

0	100	200	300	400

Significance of Overlap

P. min Clusters (columns); *S. med* Clusters (rows)

	I	II	III	IV	V
1	1.00E+00	3.27E-04	1.00E+00	1.00E+00	1.00E+00
2	1.12E-01	1.00E+00	5.32E-01	1.35E-02	1.00E+00
3	4.51E-03	1.00E+00	1.00E+00	8.63E-01	8.42E-02

Scale: *Corrected P-value*

5.00E-02	1.00E-02	1.00E-05	1.00E-08	1.00E-10

Figure S7. Comparison of *P. miniata* and *S. mediterranea* datasets. Hierarchical clustering of *P. miniata* (top left; green indicates up-regulation and purple indicates down-regulation with respect to control) and *S. mediterranea* datasets (top right; yellow indicates up-regulation and blue indicates down-regulation with respect to control), indicating the cluster number and the number of genes assigned. For each pair of clusters, the number of overlapping orthologous genes was identified (bottom left) and significance of overlap is indicated by the corrected hypergeometic p-value (bottom right). Red indicates significant (corrected p < 0.02), grey indicates no significant overlap.

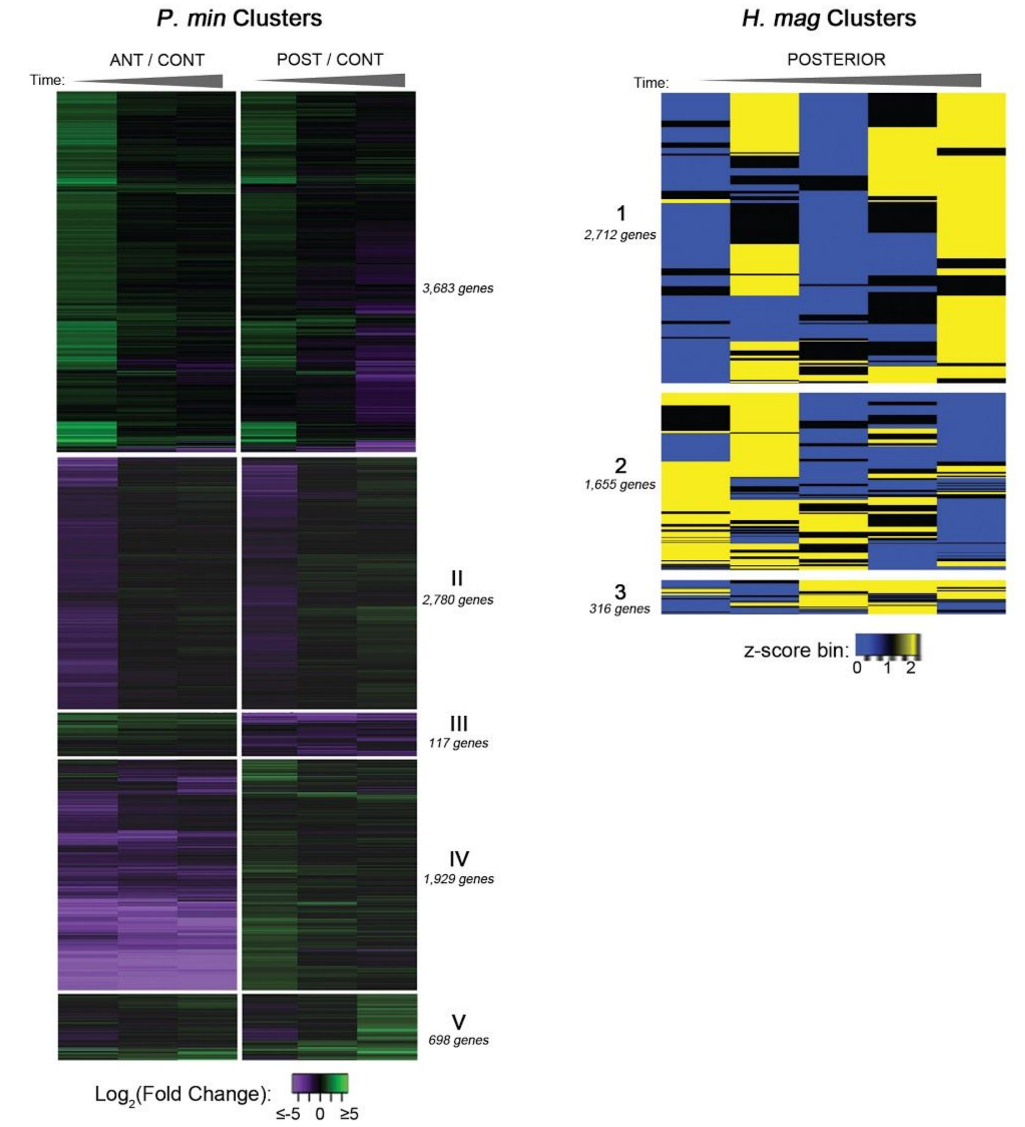

Number of Overlapping Orthologs

P. min Clusters (columns) / H. mag Clusters (rows)

H. mag Clusters	I	II	III	IV	V
1	246	390	7	67	77
2	75	318	1	62	54
3	42	47	1	17	6

1,410 total orthologs

Scale: *Number Overlapping*: 0, 100, 200, 300, 400

Significance of Overlap

P. min Clusters (columns) / H. mag Clusters (rows)

H. mag Clusters	I	II	III	IV	V
1	1.00E+00	8.72E-02	1.00E+00	2.69E-03	3.46E-01
2	8.84E-09	1.00E+00	5.39E-01	1.00E+00	1.00E+00
3	1.00E+00	4.98E-01	1.00E+00	1.00E+00	2.91E-01

Scale: *Corrected P-value*: 5.00E-02, 1.00E-02, 1.00E-05, 1.00E-08, 1.00E-10

Figure S8. Comparison of *P. miniata* and *H. magnipapillata* datasets. Hierarchical clustering of *P. miniata* (top left; green indicates up-regulation and purple indicates down-regulation with respect to control) and *H. magnipapillata* datasets (top right ; yellow indicates up-regulation and blue indicates down-regulation with respect to control), indicating the cluster number and the number of genes assigned. For each pair of clusters, the number of overlapping orthologous genes was identified (bottom left) and significance of overlap is indicated by the corrected hypergeometic p-value for the overlap is indicated (bottom right). Red indicates significant (corrected p < 0.02), grey indicates no significant overlap.

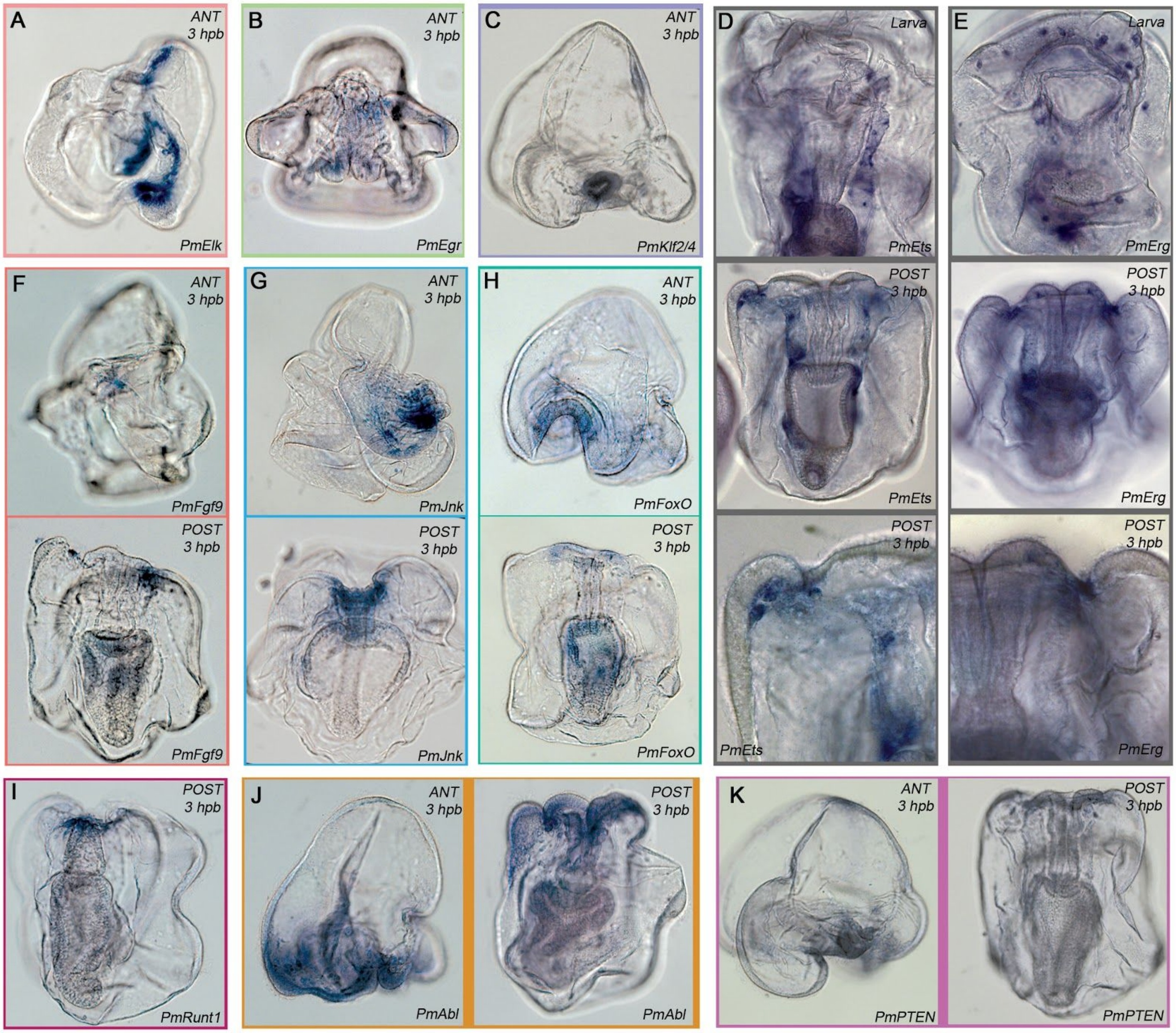

Figure S9. Additional genes that are upregulated early and localized to the wound site. Whole mount *in situ* hydridization (WMISH) in anterior fragments (ANT) at 3 hours post bisection (hpb) for genes shown in the main panel are shown in panels (A-C). Blastocoelar mesenchyme genes *Ets* (D) and *Erg* (E) are shown in both uncut larvae and regenerating posterior fragments (POST). WMISH patterns are also shown for *Fgf9* (F), *Jnk* (G), *FoxO* (H), *Runt* (I), *Abl* (J), and *Pten* (K).

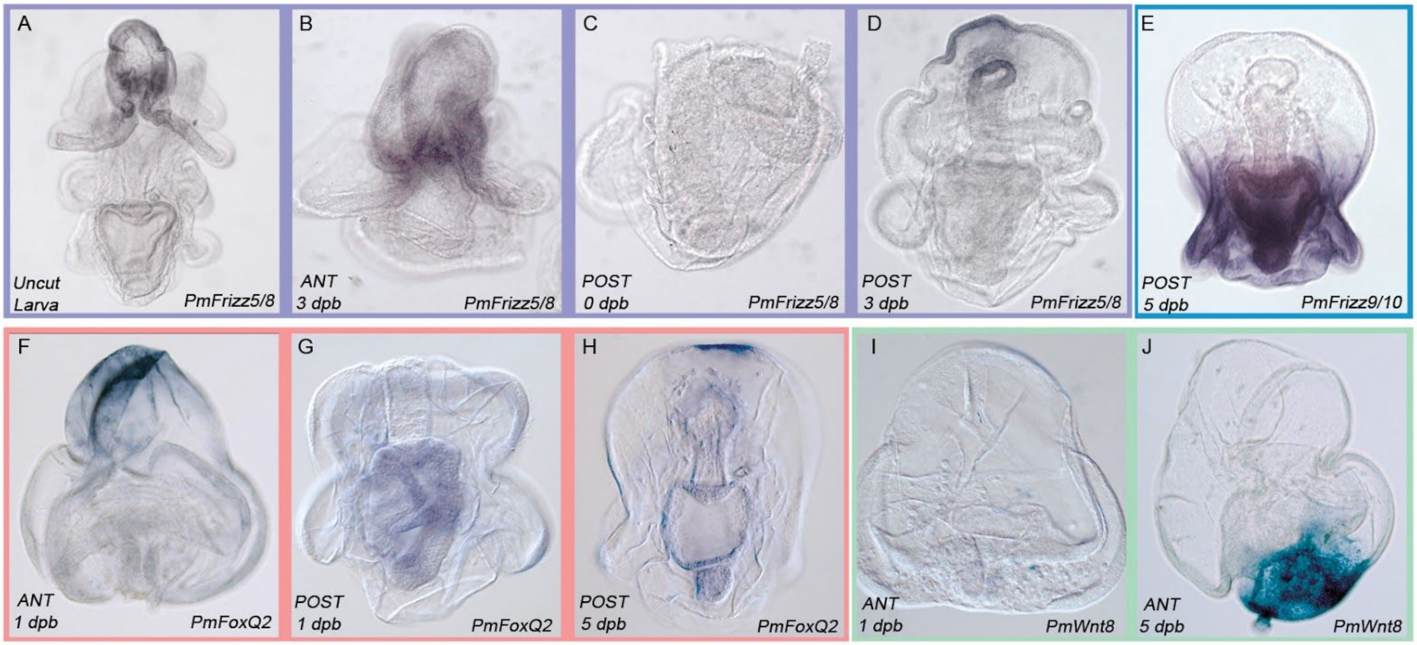

Figure S10. Recovery of anterior-posterior axis specification genes. Whole mount in situ hybridization (WMISH) illustrating the expression of *Frizz5/8* is shown in normal larvae (A) and in anterior regenerating fragments (B), but is absent from regenerating posterior fragments (C) until at least 3 days post bisection (dpb) (D). Analogous to what is shown for *Frizz5/8* in anterior fragment regeneration (B), *Frizz9/10* remains expressed in regenerating posterior fragments (E). *FoxQ2* is an anteriorly expressed gene that persists in anterior regenerating fragments (F) but is absent from posterior fragments (G) until at least 5 dpb (H), if not before. Analogously, *Wnt8* is a posterior gene whose expression is absent from anterior fragments (I), but returns by 5 dpb (J).

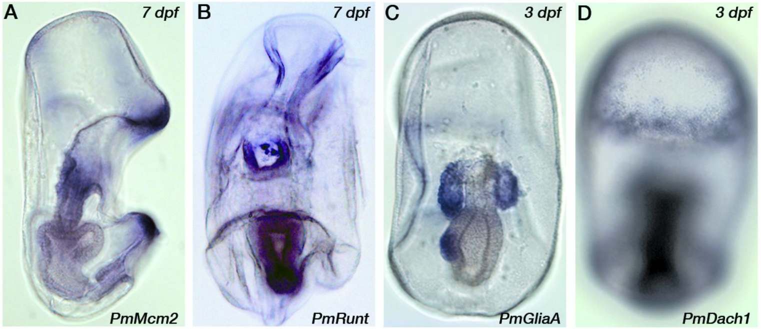

Figure S11. Whole mount *in situ* hybridization showing larval and embryonic expression of genes associated with proliferation. (A) *Mcm2* is expressed in ciliary band and foregut in normal larvae. (B) *Runt1* is normally expressed around the mouth and in the mid- and hind-gut in larvae. (C) *GliA* is expressed in coelomic epithelium in early embryos. (D) *Dach1* is expressed in ciliary band epithelium as well as presumptive gut (not shown) in early embryos.
